# Modulation of antigen delivery and lymph node activation in nonhuman primates by saponin adjuvant saponin/monophosphoryl lipid A nanoparticle

**DOI:** 10.1093/pnasnexus/pgae529

**Published:** 2024-11-25

**Authors:** Parisa Yousefpour, Yiming J Zhang, Laura Maiorino, Mariane B Melo, Mariluz A Arainga Ramirez, Sidath C Kumarapperuma, Peng Xiao, Murillo Silva, Na Li, Katarzyna K Michaels, Erik Georgeson, Saman Eskandarzadeh, Michael Kubitz, Bettina Groschel, Kashif Qureshi, Jane Fontenot, Lars Hangartner, Rebecca Nedellec, J Christopher Love, Dennis R Burton, William R Schief, Francois J Villinger, Darrell J Irvine

**Affiliations:** Koch Institute for Integrative Cancer Research, Massachusetts Institute of Technology, Cambridge, MA 02139, USA; Ragon Institute of Massachusetts General Hospital, Massachusetts Institute of Technology and Harvard University, Cambridge, MA 02139, USA; Consortium for HIV/AIDS Vaccine Development, The Scripps Research Institute, La Jolla, CA 92037, USA; Koch Institute for Integrative Cancer Research, Massachusetts Institute of Technology, Cambridge, MA 02139, USA; Ragon Institute of Massachusetts General Hospital, Massachusetts Institute of Technology and Harvard University, Cambridge, MA 02139, USA; Consortium for HIV/AIDS Vaccine Development, The Scripps Research Institute, La Jolla, CA 92037, USA; Department of Biological Engineering, Massachusetts Institute of Technology, Cambridge, MA 02139, USA; Koch Institute for Integrative Cancer Research, Massachusetts Institute of Technology, Cambridge, MA 02139, USA; Ragon Institute of Massachusetts General Hospital, Massachusetts Institute of Technology and Harvard University, Cambridge, MA 02139, USA; Consortium for HIV/AIDS Vaccine Development, The Scripps Research Institute, La Jolla, CA 92037, USA; Howard Hughes Medical Institute, Chevy Chase, MD 20815, USA; Koch Institute for Integrative Cancer Research, Massachusetts Institute of Technology, Cambridge, MA 02139, USA; Ragon Institute of Massachusetts General Hospital, Massachusetts Institute of Technology and Harvard University, Cambridge, MA 02139, USA; Consortium for HIV/AIDS Vaccine Development, The Scripps Research Institute, La Jolla, CA 92037, USA; New Iberia Research Center, University of Louisiana at Lafayette, New Iberia, LA 70560, USA; Research Imaging Institute, University of Texas Health San Antonio, San Antonio, TX 78229, USA; New Iberia Research Center, University of Louisiana at Lafayette, New Iberia, LA 70560, USA; Koch Institute for Integrative Cancer Research, Massachusetts Institute of Technology, Cambridge, MA 02139, USA; Ragon Institute of Massachusetts General Hospital, Massachusetts Institute of Technology and Harvard University, Cambridge, MA 02139, USA; Consortium for HIV/AIDS Vaccine Development, The Scripps Research Institute, La Jolla, CA 92037, USA; Koch Institute for Integrative Cancer Research, Massachusetts Institute of Technology, Cambridge, MA 02139, USA; Koch Institute for Integrative Cancer Research, Massachusetts Institute of Technology, Cambridge, MA 02139, USA; Ragon Institute of Massachusetts General Hospital, Massachusetts Institute of Technology and Harvard University, Cambridge, MA 02139, USA; Consortium for HIV/AIDS Vaccine Development, The Scripps Research Institute, La Jolla, CA 92037, USA; Howard Hughes Medical Institute, Chevy Chase, MD 20815, USA; Ragon Institute of Massachusetts General Hospital, Massachusetts Institute of Technology and Harvard University, Cambridge, MA 02139, USA; Consortium for HIV/AIDS Vaccine Development, The Scripps Research Institute, La Jolla, CA 92037, USA; Department of Immunology and Microbiology, The Scripps Research Institute, La Jolla, CA 92037, USA; Ragon Institute of Massachusetts General Hospital, Massachusetts Institute of Technology and Harvard University, Cambridge, MA 02139, USA; Consortium for HIV/AIDS Vaccine Development, The Scripps Research Institute, La Jolla, CA 92037, USA; Department of Immunology and Microbiology, The Scripps Research Institute, La Jolla, CA 92037, USA; Ragon Institute of Massachusetts General Hospital, Massachusetts Institute of Technology and Harvard University, Cambridge, MA 02139, USA; Consortium for HIV/AIDS Vaccine Development, The Scripps Research Institute, La Jolla, CA 92037, USA; Department of Immunology and Microbiology, The Scripps Research Institute, La Jolla, CA 92037, USA; Ragon Institute of Massachusetts General Hospital, Massachusetts Institute of Technology and Harvard University, Cambridge, MA 02139, USA; Consortium for HIV/AIDS Vaccine Development, The Scripps Research Institute, La Jolla, CA 92037, USA; Department of Immunology and Microbiology, The Scripps Research Institute, La Jolla, CA 92037, USA; Koch Institute for Integrative Cancer Research, Massachusetts Institute of Technology, Cambridge, MA 02139, USA; New Iberia Research Center, University of Louisiana at Lafayette, New Iberia, LA 70560, USA; Consortium for HIV/AIDS Vaccine Development, The Scripps Research Institute, La Jolla, CA 92037, USA; Department of Immunology and Microbiology, The Scripps Research Institute, La Jolla, CA 92037, USA; Department of Immunology and Microbiology, The Scripps Research Institute, La Jolla, CA 92037, USA; Koch Institute for Integrative Cancer Research, Massachusetts Institute of Technology, Cambridge, MA 02139, USA; Ragon Institute of Massachusetts General Hospital, Massachusetts Institute of Technology and Harvard University, Cambridge, MA 02139, USA; Department of Chemical Engineering, Massachusetts Institute of Technology, Cambridge, MA 02139, USA; Ragon Institute of Massachusetts General Hospital, Massachusetts Institute of Technology and Harvard University, Cambridge, MA 02139, USA; Consortium for HIV/AIDS Vaccine Development, The Scripps Research Institute, La Jolla, CA 92037, USA; Department of Immunology and Microbiology, The Scripps Research Institute, La Jolla, CA 92037, USA; IAVI Neutralizing Antibody Center, The Scripps Research Institute, La Jolla, CA 92037, USA; Ragon Institute of Massachusetts General Hospital, Massachusetts Institute of Technology and Harvard University, Cambridge, MA 02139, USA; Consortium for HIV/AIDS Vaccine Development, The Scripps Research Institute, La Jolla, CA 92037, USA; Department of Immunology and Microbiology, The Scripps Research Institute, La Jolla, CA 92037, USA; IAVI Neutralizing Antibody Center, The Scripps Research Institute, La Jolla, CA 92037, USA; Moderna Inc., Cambridge, MA 02139, USA; New Iberia Research Center, University of Louisiana at Lafayette, New Iberia, LA 70560, USA; Department of Biology, University of Louisiana at Lafayette, New Iberia, LA 70560 USA; Koch Institute for Integrative Cancer Research, Massachusetts Institute of Technology, Cambridge, MA 02139, USA; Ragon Institute of Massachusetts General Hospital, Massachusetts Institute of Technology and Harvard University, Cambridge, MA 02139, USA; Consortium for HIV/AIDS Vaccine Development, The Scripps Research Institute, La Jolla, CA 92037, USA; Department of Biological Engineering, Massachusetts Institute of Technology, Cambridge, MA 02139, USA; Howard Hughes Medical Institute, Chevy Chase, MD 20815, USA; Department of Chemical Engineering, Massachusetts Institute of Technology, Cambridge, MA 02139, USA; Department of Materials Science and Engineering, Massachusetts Institute of Technology, Cambridge, MA 02139, USA

**Keywords:** adjuvant, HIV, nonhuman primate, SMNP (saponin/MPLA nanoparticles), vaccine

## Abstract

Saponin-based vaccine adjuvants are potent in preclinical animal models and humans, but their mechanisms of action remain poorly understood. Here, using a stabilized HIV envelope trimer immunogen, we carried out studies in nonhuman primates (NHPs) comparing the most common clinical adjuvant aluminum hydroxide (alum) with saponin/monophosphoryl lipid A nanoparticles (SMNP), an immune-stimulating complex–like adjuvant. SMNP elicited substantially stronger humoral immune responses than alum, including 7-fold higher peak antigen-specific germinal center B-cell responses, 18-fold higher autologous neutralizing antibody titers, and higher levels of antigen-specific plasma and memory B cells. Positron emission tomography and computed tomography imaging in live NHPs showed that, unlike alum, SMNP promoted rapid antigen accumulation in both proximal and distal lymph nodes (LNs). SMNP also induced strong type I interferon transcriptional signatures, expansion of innate immune cells, and increased antigen-presenting cell activation in LNs. These findings indicate that SMNP promotes multiple facets of the early immune response relevant for enhanced immunity to vaccination.

**Significance Statement**

Saponins are an important class of vaccine adjuvants. Here, we show that a nanoparticle-forming saponin-based adjuvant termed SMNP (saponin/monophosphoryl lipid A nanoparticles) elicits higher levels of germinal center B cells, neutralizing antibodies, and memory B cells than aluminum hydroxide in nonhuman primates (NHPs). Using whole-animal imaging of vaccine biodistribution in NHPs, we uncover a unique vaccine adjuvant mechanism of action for SMNP, which we show facilitates rapid antigen accumulation in multiple lymph nodes draining the immunization site, accompanied by robust innate immune activation. These findings provide important new insights into this promising saponin adjuvant and uncover a novel mechanism of action for vaccine adjuvants based on promoting antigen accumulation in secondary lymphoid tissues distal from the immunization site.

## Introduction

Adjuvants are essential for shaping the immune responses to subunit vaccines (1, 2). The development of vaccines capable of neutralizing highly mutable pathogens may require adjuvants that can robustly promote germinal center (GC) responses, where B-cell selection and affinity maturation occur (3–6). To date, only a small number of adjuvants have been licensed for prophylactic vaccines in humans, and there is great interest in developing new vaccine adjuvants that can enhance GC responses, promote greater breadth of humoral immunity, or elicit broadly neutralizing antibody (bnAb) responses of greater magnitude and durability. Effective adjuvants may be particularly critical for promoting the development of bnAbs against HIV, influenza, sarbecoviruses, and other emerging pathogens.

Saponins are one particularly potent class of adjuvants. Saponins are triterpene glycosides first studied as natural products isolated from the bark of the *Quillaja saponaria* tree. Saponins have surfactant-like activity and are typically formulated with lipids and cholesterol to avoid toxicity associated with membrane disruption caused by free saponin (7). Examples of licensed vaccines employing saponin adjuvants include Shingrix and Mosquirix, which employ Glaxo Smith Kline's AS01 adjuvant (1, 8, 9) (a liposomal formulation of saponin and the Toll-like receptor [TLR]-4 agonist monophosphoryl lipid A [MPLA]), and the Novavax COVID-19 vaccine, which employs Matrix M adjuvant (10). Matrix M is an example of an immune-stimulating complex (ISCOM) formulation of saponins, wherein saponins self-assemble with cholesterol and phospholipids to form ∼40 nm diameter cage-like nanoparticles. The precise mechanisms of action underlying the potency of saponin adjuvants remain poorly understood, though preclinical studies have demonstrated that saponins activate inflammasomes, promote antigen cross-presentation by dendritic cells (DCs), and trigger production of inflammatory cytokines and chemokines (11–17).

We recently described a novel saponin adjuvant formed by physically incorporating the lipid TLR4 agonist MPLA into ISCOMs, forming saponin/MPLA nanoparticles (SMNP) (11, 18). SMNP was found to prime extremely strong GC, T follicular helper (Tfh), and antibody responses in mice. Mechanistically, we found that in mice, SMNP enhanced lymph flow and the entry of antigens into draining lymph nodes (dLNs), accompanied by robust induction of inflammatory cytokines and chemokines in dLNs (11). In a pilot study employing a stabilized HIV envelope (Env) trimer immunogen in rhesus macaques (RMs), SMNP elicited stronger antibody titers, antibody effector functions, and tier 2 neutralizing antibody responses than two other experimental adjuvants of interest, a lipid-conjugated CpG TLR-9 agonist and a STING agonist (11). These preclinical experiments employed SMNP prepared using Quil-A saponin, but based on the promising preclinical data, SMNP has been manufactured using highly purified QS-21 saponin for an ongoing first-in-humans clinical trial (HVTN144). However, it has remained unclear how QS-21-based SMNP would compare with the clinical gold standard adjuvant aluminum hydroxide (hereafter alum for simplicity), and whether the mechanisms of action identified in rodents would also be relevant in a large animal model closer to humans.

Here, we compared immune responses and mechanisms of action following immunization of RMs with a stabilized HIV Env trimer immunogen termed MD39 (19), adjuvanted either by alum (Alhydrogel) or QS-21 SMNP (hereafter SMNP for simplicity). We found that SMNP elicited substantially stronger GC and Tfh responses than alum, as well as increased autologous neutralizing antibody titers. To gain insight into how the choice of adjuvant influenced early events in dLNs, we carried out studies combining positron emission tomography (PET) imaging, flow cytometry, and single-cell RNA sequencing (scRNA-seq) to evaluate antigen trafficking, immune cell recruitment and phenotype, and transcriptional status of immune cells in dLNs. Mirroring findings in rodents, these experiments revealed that, unlike alum, SMNP promoted antigen delivery to both proximal and more distal LNs, triggered a strong type I interferon (IFN) response in dLNs, and elicited changes in innate immune cells in both proximal and more distal LNs of nonhuman primates (NHPs).

## Results

### SMNP elicits much stronger GC responses than alum

**Fig. 1.**
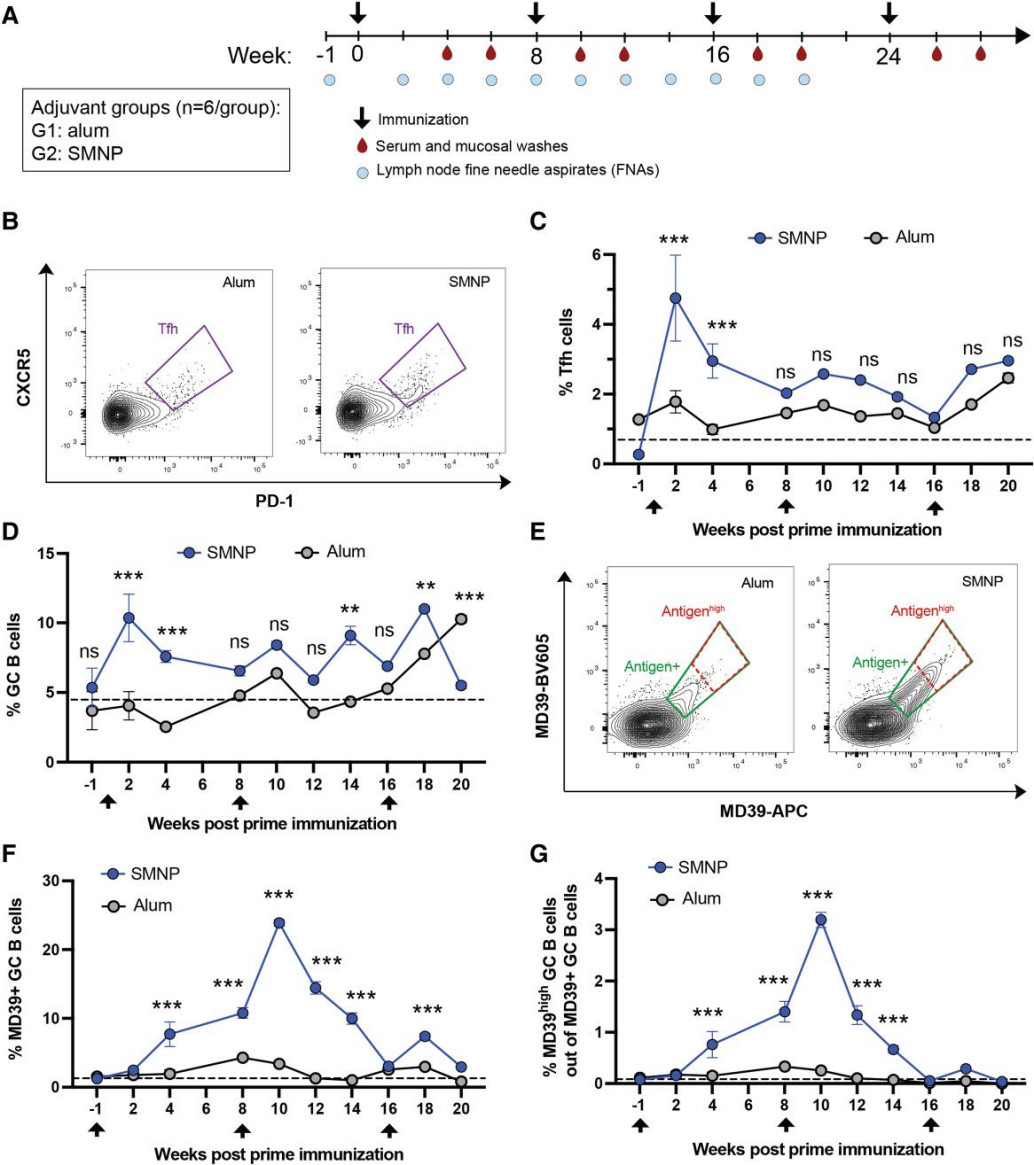
SMNP promotes stronger Tfh and GC responses than alum. RMs (*n* = 6/group) were vaccinated s.c. bilaterally in the inner thighs, with 50 µg MD39 trimer combined with either 500 µg alum or 188 µg SMNP per site at weeks 0, 8, 16, and 24. A) Study diagram. B–G) Tfh and GC responses detected by flow cytometry analysis of LN FNAs. B) Representative Tfh flow cytometry plots gated on CD4^+^ T cells. C, D) Frequencies of GC Tfh cells (CD3^+^CD4^+^PD1^+^ CXCR5^+^, C) and GC B cells (CD20^+^BCL6^+^Ki67^+^, D) over time. E) Representative flow cytometry plots of trimer-binding GC B cells, indicating total antigen^+^ (solid rectangle) and antigen^high^ (dashed rectangle) gates. F and G) Frequencies of total (F) and high-binding (G) antigen-specific GC B cells. Dashed lines show baseline levels calculated from the responses at week −1. Statistical analyses were performed using one-way ANOVA, followed by Sidak's multiple comparisons post hoc test. *P < 0.05, **P < 0.01, ***P < 0.001.

We first carried out an immunogenicity study in RMs comparing alum with SMNP adjuvant. SMNP particles were synthesized incorporating QS-21 saponin, which is the highly purified saponin fraction employed in clinical saponin adjuvant formulations, to mimic the SMNP composition entering clinical testing (3, 13). These adjuvants were combined with a highly stabilized native-like SOSIP HIV Env trimer termed MD39, representative of “polishing” immunogens in development for HIV vaccine regimens targeting the development of bnAbs (19). Two groups of RMs were immunized subcutaneously in the left and right inner thighs with MD39 trimer formulated with either alum or SMNP, administered at weeks 0, 8, 16, and 24 (Fig. 1A). We opted to test this multiboost immunization scheme motivated in part because we expected SMNP would be more potent than alum, but we wanted to determine whether humoral responses elicited by alum could “catch up” to SMNP by simply increasing the number of immunizations. We first analyzed the kinetics of the GC responses by fine-needle aspirate (FNA) sampling of draining inguinal LNs over the first 20 weeks of the study (Fig. S1). Previous studies have shown that FNAs are well tolerated and provide a representative sample of the cellular composition of the entire LN (20). Alum immunization showed limited, if any, detectable Tfh expansion following each of the first three immunizations (Fig. 1B and C). In contrast, SMNP elicited a pronounced expansion of Tfh cells at 2 weeks postprime immunization (WPPI) to a level ∼2.7-fold greater than the alum group, which contracted by week 6, followed by less prominent changes at the subsequent boosts (Fig. 1B and C). Similar trends were observed for total GC B cells over time: GC cells expanded to a peak of ∼10% of all B cells 2 WPPI for SMNP (Fig. 1D). Interestingly, alum-induced GCs only began expanding after the third immunization (Fig. 1D). Antigen-binding GC B cells were also assessed by staining B cells with tetramers of MD39 labeled with two different dyes (Fig. 1E), and here the differences in GC responses were particularly striking: alum immunization elicited weak trimer-binding GC responses that never exceeded 5% of the total GC B-cell response, while SMNP elicited a trimer-specific GC B population that peaked at ∼11% of all GC B cells postprime, and further expanded to ∼25% of all GC B cells following the first boost at week 8 (Fig. 1F). Gating on GC B cells that stained brightly with antigen as a proxy for high antigen affinity (Antigen^high^ cells, Fig. 1E), SMNP elicited a peak response where ∼3% of the trimer-specific cells were in this high-affinity gate (Fig. 1G). Both the total GC response and trimer-specific response failed to expand as greatly following the third immunization, which we suspect reflects the high titers of antibody already present in the animals at the time of the third injection. Altogether, these data indicated that SMNP is capable of eliciting strongly enhanced GC responses in comparison with alum.

### SMNP primes higher antibody and bone marrow plasma cell responses than alum even following repeat dosing

We next evaluated early serum cytokine and plasmablast responses, serum antibody levels, and neutralizing antibody titers in the NHPs. Following the primary immunization and the first boost, we collected blood samples serially over 72 h to monitor for elevations of systemic cytokines/chemokines. Alum immunization elicited very low levels of cytokines or chemokines postprime or postboost (Fig. S2). In contrast, SMNP vaccination elicited transient spikes in proinflammatory cytokines interleukin (IL)-6, CXCL10, and MCP1, and antiinflammatory cytokine IL-10, which peaked by 24 h and returned to baseline by 72 h; IL-6 was particularly elevated following the boost (Fig. S2). Transient inflammatory cytokine signatures in the blood are typical of strong adjuvants and are also reported in humans for saponin adjuvants (21, 22). IL-10 is known to be induced by IL-6 as part of a counter-regulatory antiinflammatory response in humans (23), and elevated IL-10 levels have also been observed in clinical studies of GSK's AS01b adjuvant, which contains saponins and induces similar transient elevations of IL-6 following vaccination (21).

**Fig. 2.**
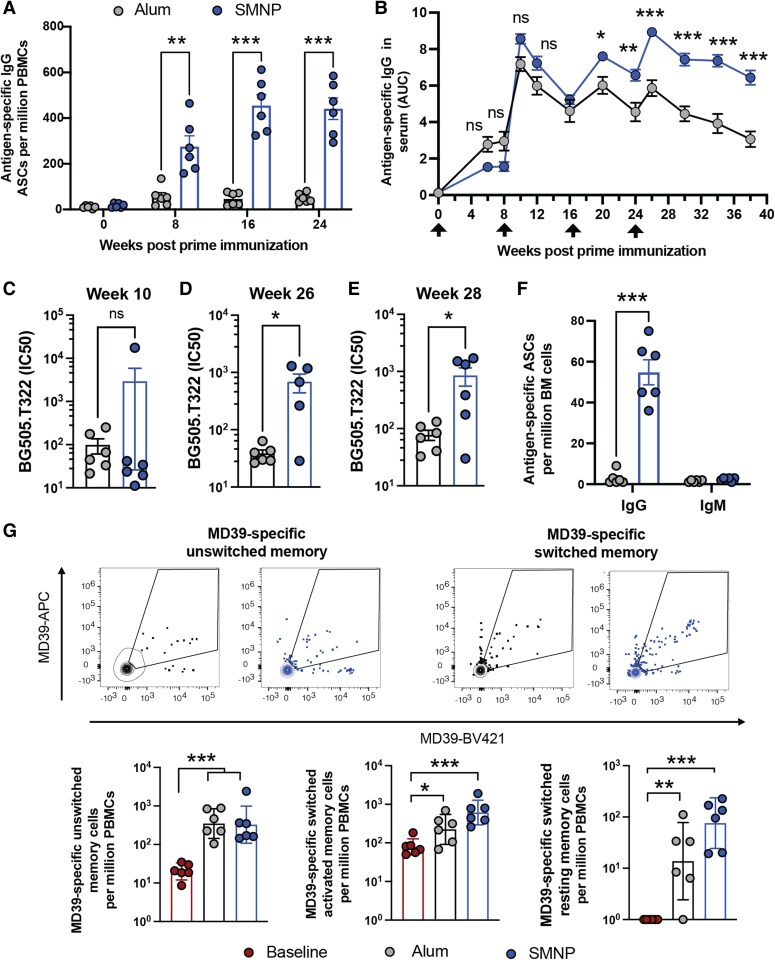
SMNP primes stronger plasmablast, serum antibody, and neutralizing antibody responses than alum. A) Frequency of circulating antigen-specific antibody-secreting plasmablasts detected in PBMCs by ELISpot. B) Serum IgG responses quantified as area under the curve (AUC) of ELISA optical density vs. serum dilution curves. C–E) Autologous tier 2 neutralizing antibody titers at weeks 10 (C), 26 (D), and 28 (E) PPI. F) Trimer-specific IgG or IgM antibody-secreting BM plasma cells were quantified at week 62 by ELISpot. G) Antigen-specific memory B cells among PBMCs at week 34 postvaccination. Shown are representative flow cytometry contour plots of antigen-specific cells gated on unswitched and switched memory B cells (top panels) and frequency of respective unswitched and switched (resting or activated) memory B cells (bottom panels). Data are presented as geometric mean ± 95% CI. Statistical comparisons were performed using one-way ANOVA, followed by Sidak's (A, B) or Tukey's post hoc test (G) or Student's t test (C, D, E, F). *P < 0.05; **P < 0.01; ***P < 0.001. Log-distributed datasets were log-transformed before statistical analysis.

Antigen-specific plasmablasts in the peripheral blood were analyzed via a B-cell ELISpot assay 6 days post each immunization. Weak antigen-specific IgG responses were detected following each immunization with alum, while SMNP induced robust priming of MD39-specific IgG plasmablasts following each booster immunization (Fig. 2A). Both alum and SMNP immunizations primed high levels of MD39 trimer-binding serum IgG, but SMNP responses were higher, especially following the final boost at week 24 (Fig. 2B). Serum IgA levels were, however, very low for both immunizations (Fig. S3A). Transient spikes in trimer-specific IgG were detected in nasal and vaginal washes post boost immunizations that were not sustained, suggesting this was transudated antibody driven by peak antibody production post boost (Fig. S3B–G). HIV autologous tier 2 neutralizing antibody titers were assessed after the first boost and after the final boost. Little neutralization was detected in either group after the first boost, but at week 26, trimer immunization with SMNP had elicited mean neutralizing titers ∼1,000, and these neutralizing titers remained ∼10-fold greater than that elicited by alum immunization at week 28 (Fig. 2C–E).

Two key cellular outputs of the humoral response to vaccination are memory B cells and long-lived plasma cells. We assessed plasma cell development by quantifying bone marrow (BM)-resident antibody-secreting cells (ASCs) secreting different antibody isotypes by ELISPOT at week 62. While alum elicited very low frequencies of trimer-specific ASCs, high levels of IgG-secreting plasma cells were detected in the SMNP group (Fig. 2F). Flow cytometry analysis of peripheral blood mononuclear cells (PBMCs) at week 34 to detect trimer-binding memory B cells revealed significant populations of class-switched resting and activated MD39-specific B cells (Fig. S4) (24), which trended higher for the SMNP-vaccinated animals (Fig. 2G). Thus, SMNP appears to be more effective than alum in promoting multiple facets of the humoral response to HIV Env immunogens.

### SMNP promotes antigen distribution to many LNs in the draining lymphatic chain

**Fig. 3.**
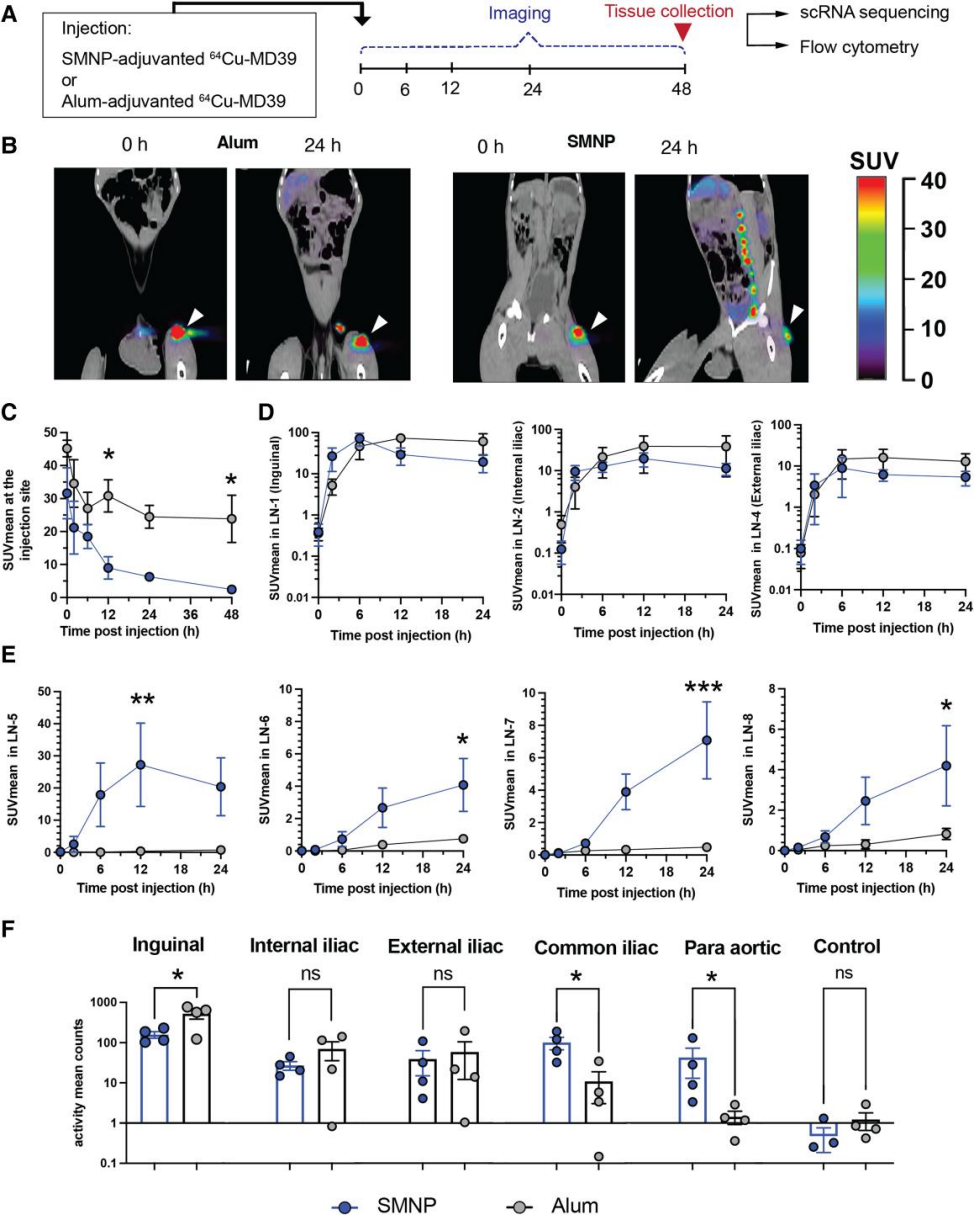
SMNP promotes antigen delivery to more distal LNs than alum. NHPs (*n* = 4 animals/group) were immunized s.c. in the left inner thigh with 50 µg ^64^Cu-labeled MD39 trimer combined with either 500 µg alum or 188 µg SMNP, and then imaged over time by PET/CT scanning. A) Timeline for PET/CT imaging studies. B) Pseudocolor PET signal overlaid on grayscale CT scans at 0 and 24 h postimmunization. White arrows indicate injection site. C–E) Radiolabeled MD39 signal from PET scan quantified as SUVmean of gated ROIs at 0, 6, 12, 24, and 48 h postinjection. Shown are SUVmean of antigen signal over time at the injection site (C), most proximal dLNs (D), and more distal LNs in the ipsilateral draining lymphatic chain, numbered in order from nearest to farthest from the injection site (E). F) SUVmean of PET antigen signal measured in the indicated LNs ex vivo following tissue collection 48 h postinjection. Data are presented as mean ± SEM. Statistical analyses were performed using one-way ANOVA, followed by Sidak's post hoc test (C–E) or Student's t test (F). *P < 0.05, **P < 0.01, ***P < 0.001.

Based on the findings from the immunogenicity study, we sought to gain insights into why SMNP was so much more effective in driving the humoral response than alum. In mice, we found that SMNP induced lymph vessel swelling, enhanced antigen drainage through lymph, and increased antigen entry into dLNs, leading to priming of GC responses in both proximal and secondary dLNs (11). To determine whether altered antigen trafficking could also underlie the efficacy of SMNP in NHPs, we carried out a study to visualize antigen dissemination from the injection site following alum or SMNP immunization using PET imaging in RMs. MD39 was conjugated with the radiometal chelator 1,4,7,10-tetraazacyclododecane-1,4,7,10-tetraacetic acid (DOTA), to enable ^64^Cu labeling of the protein. Groups of macaques were then immunized s.c. in the left inner thigh with ^64^Cu-MD39 trimer mixed with alum or SMNP, and imaged serially over time by PET with tandem X-ray computed tomography (CT; Fig. 3A). Co-registered PET/CT images revealed that after 24 h, antigen was primarily localized at the injection site and the most proximal 2–3 draining inguinal and iliac LNs for alum/MD39-immunized animals (Fig. 3B, left). In striking contrast, by 24 h, much of the antigen in the trimer/SMNP-immunized animals had left the injection site and accumulated in a series of 6–8 LNs in the ipsilateral draining lymphatic chain (Fig. 3B, right, and Fig. S5). Quantitation of the mean standard uptake values (SUVmean) at the site of injection (SOI) showed almost complete clearance of antigen in the SMNP group by 48 h, while ∼50% of the antigen was still retained at the SOI in the alum group at this time point (Fig. 3C).

Examining antigen uptake in the ipsilateral dLN chain, we numbered the LNs from 1 (nearest to the injection site, the inguinal LN) to 8 (most distal LN) and tracked the SUVmean over time for the nodes that could be definitively identified in animals over time. Antigen levels were highest in the three most proximal nodes (inguinal, internal iliac, and external iliac), and not statistically different for alum vs. SMNP (Fig. 3D). However, in the more distal nodes, significant antigen uptake was still detected for trimers administered with SMNP, whereas negligible levels of signal were detected for trimers administered with alum (Fig. 3E). At 48 h the animals were sacrificed and LNs were collected for confirmation of signal by ex vivo PET scanning (Fig. 3A); this analysis showed the same trend as the in vivo imaging, with the inguinal, internal iliac, and external iliac nodes acquiring similar levels of antigen for both alum and SMNP, while the next two more distal nodes (common iliac and paraortic) had 10-fold or more antigen signal in the SMNP group vs. the alum group (Fig. 3F). (More distal nodes were too difficult to locate in the necropsy for further analysis.) Altogether, these data suggest that, similar to observations in mice, SMNP promotes antigen trafficking to multiple LNs, and antigen is seen reaching a series of nodes extending well beyond those most proximal to the injection site.

### SMNP induces a strong type I IFN signature in draining LNs

**Fig. 4.**
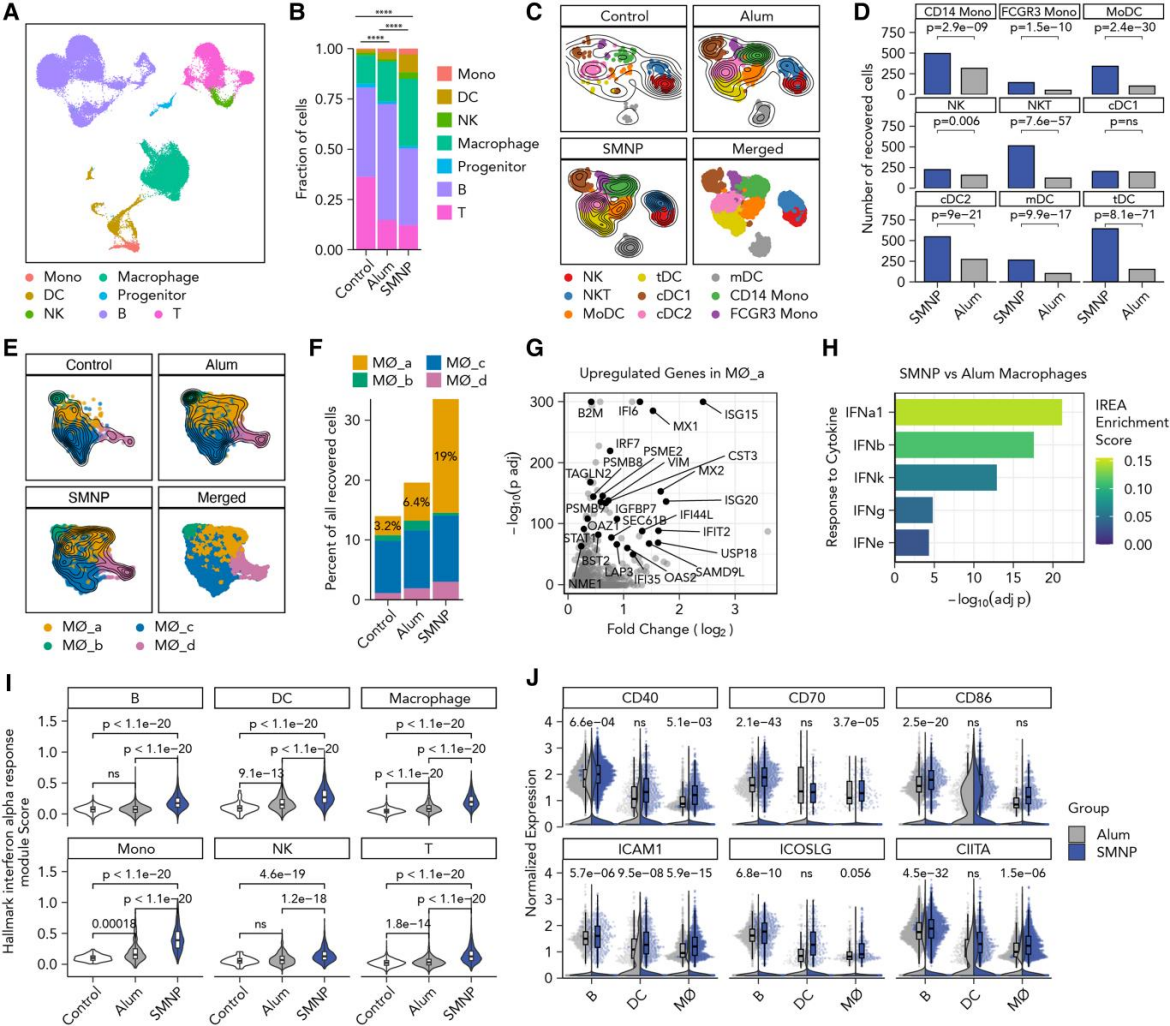
SMNP induces a strong type I IFN signature in dLNs. A) UMAP of recovered cell lineages. B) Distribution of recovered cell lineages in control contralateral (*n*_cell_ = 8,785, *n*_sample_ = 1), alum (*n*_cell_ = 22,422, *n*_sample_ = 3), and SMNP (*n*_cell_ = 22,401, *n*_sample_ = 3) dLNs. *P*-values were calculated using a two-tailed χ^2^ test with Bonferroni post hoc correction. ****P < 0.0001. C) UMAP of cell phenotypes among monocytes, DCs, and NK cells. D) Bar plots of the numbers of recovered cells between SMNP and alum for NK, DC, and monocyte phenotypes. *P*-values were calculated from Fisher's exact test with Bonferroni post hoc correction. E) UMAP of phenotypic clusters of macrophages (MØs). F) Percent of recovered macrophage clusters per group. For (A, C, and E), the contour lines represent the density of overlapping cells. G) Volcano plot of significantly up-regulated genes in the MØ_a cluster compared with the rest of the macrophage clusters. H) Bar plot of significantly enriched cytokine responses in SMNP macrophages compared with alum macrophages based on the IREA. I) The cellular module scores for the Hallmark IFN-α response gene set of individual cells from each group per cell lineage. *P*-values were calculated using the Kruskal–Wallis test and Dunn's post hoc test with Bonferroni post hoc correction. J) The expression levels of representative co-stimulatory genes in APCs (macrophage, DC, B cell) from SMNP (blue) and alum (gray) groups. The violin plots show the overall density of expression levels. The jittered dots represent individual cells and their expression levels. The boxplots show the distribution of cells with nonzero expression. *P*-values were calculated using all cells with the Wilcoxon rank sum test with Bonferroni post hoc correction.

We next sought to assess changes in LN activation induced by SMNP vs. alum in the proximal inguinal dLNs. LNs from three NHPs per group were collected following PET imaging at 48 h postimmunization and were processed for scRNA-seq analysis (Fig. 3A). A contralateral inguinal LN without antigen signal from an NHP immunized with alum was collected as the control. Single cells, which were CD3^low^CD8^low^CD20^low^, were flow sorted to moderately enrich for cells of myeloid lineage using flow cytometry (Fig. S6A). After quality control of the scRNA-seq data, we identified seven cell lineages, including natural killer (NK) cells, monocytes, macrophages, DCs, T cells, B cells, and CD34^+^ hematopoietic progenitor cells, and recovered 8,785, 22,422, and 22,401 cells from the LNs of the control, alum, and SMNP groups, respectively (Figs. 4A and B, S6B).

For this very early time point, we focused on the analysis of innate cells. We identified two clusters of monocytes, including *CD14*^high^ monocytes and CD16(*FCGR3*)^high^ monocytes, two clusters of NK cells, one of which is *CD3^high^KLRB1^low^* NKT like, five clusters of DCs including migratory DCs with high expression of *CCR7*, *FSCN1*, *MARCKSL1*, and *LAMP3* (25), type 1 conventional DCs (cDC1), expressing *CLEC9A*, *CADM1*, *BATF3*, *XCR1*, *BTLA* (26–28), type 2 conventional DCs (cDC2) with canonical markers of *CD1C*, *FCER1A*, *CLEC10A*, and *CLEC4A* (26–28), monocyte-derived DCs (MoDC), expressing *NCOR*, *ZEB2*, and *SLC8A1* (29–31), and recently identified *AXL*^+^ transitional DCs (tDCs) (32–36), which are effective at priming CD4^+^ T cells, with a continuum of cDC2 and plasmacytoid DC-lineage markers, such as *TCF4*, *CD141(THBD)*, *SPIB*, *IRF7*, *BCL11A*, and *PPP1R14A* (Figs. 4C and S6C). Except for cDC1 cells, cell counts and frequencies of each innate cell population were significantly increased in SMNP LNs compared with alum LNs (Figs. 4D and S6D). Particularly, 4.3-, 3.4-, and 4.3-fold more NK T cells, MoDCs, and tDCs were found in SMNP LNs (Fig. 4D).

We next focused on macrophages, which increased in frequency moving from control to alum to SMNP LNs (Fig. 4B). Four phenotypic clusters were identified, and one of these, MØ_a cell, was present at 3- and ∼6-fold higher frequencies in the SMNP group vs. alum and control LNs, respectively (Fig. 4E and F). Differential gene expression analysis of MØ_a cells vs. the other macrophage clusters showed that the former up-regulated IFN-responsive genes, such as *ISG15*, *MX1*, *IFI6*, and *B2M* (37) (Fig. 4G). This suggested that SMNP polarized macrophages more strongly toward a type I IFN-activated state than alum. To verify if this shift in phenotype was significantly different in SMNP vs. alum LNs, we performed a pseudo-bulk differential gene expression analysis between all macrophages from the SMNP vs. alum groups and identified 764 genes significantly up-regulated (adjusted *P* < 0.01) in SMNP macrophages (Fig. S6E). We next performed immune response enrichment analysis (IREA) (38), where the up-regulated genes in all macrophages from SMNP-vaccinated LNs were compared with gene signatures of macrophages polarized with 86 individual cytokines. We found that SMNP-treated macrophages were significantly enriched with gene signatures of response to IFN-α1, IFN-β, IFN-κ, IFN-γ, and IFN-3 cytokines (Fig. 4H), indicating that SMNP indeed polarized macrophages toward an IFN-stimulated transcriptional state more strongly than alum.

To determine whether type I IFN stimulation signatures were expressed in other cells isolated from SMNP-immunized LNs, we scored the expression profile of each recovered cell by the Hallmark Interferon Alpha Response gene set from the Molecular Signatures Database (39). Strikingly, this analysis revealed that cells across all cell lineages up-regulated genes involved in the type I IFN response (Fig. 4I). To see whether these gene expression signatures extended to lymphocytes, we also analyzed the transcriptional phenotypes of B and T cells. We identified eight phenotypic clusters of B cells (Fig. S7A and B) and five clusters of T cells (Fig. S7C and D). Among the B- and T-cell clusters, we observed differences in the phenotypic composition between alum and SMNP and were able to identify clusters MBC_c and T_c of B and T cells, respectively, which were enriched in the SMNP group (Fig. S7E and F). Both MBC_c and T_c cells showed strong type I IFN response signatures, such as *ISG15*, *ISG20*, *MX1*, *IFI27*, *IRF9*, *B2M*, etc. (Fig. S7G and H), corroborating that SMNP induced a broad IFN-stimulated state in LN cells.

Type I IFN signaling has been shown to enhance DC activation and differentiation (40), improve T-cell priming and the development of Tfh cells (41), and promote B-cell survival and differentiation into ASCs (42, 43). Consistent with the literature, we found significantly increased expression of co-stimulatory molecules, such as CD40, CD70, CD86, ICAM1, ICOSLG, and CIITA (the activator of MHC class II gene transcription) in antigen-presenting cells (APCs) from SMNP-vaccinated LNs compared with alum-vaccinated LNs (Fig. 4J). Collectively, these results suggest that relative to alum, SMNP rapidly increased the frequency of diverse innate immune cells, triggered a broad type I IFN response, and promoted activation of APCs in dLNs.

### Innate immune activation induced by SMNP vs. alum in draining LNs

**Fig. 5.**
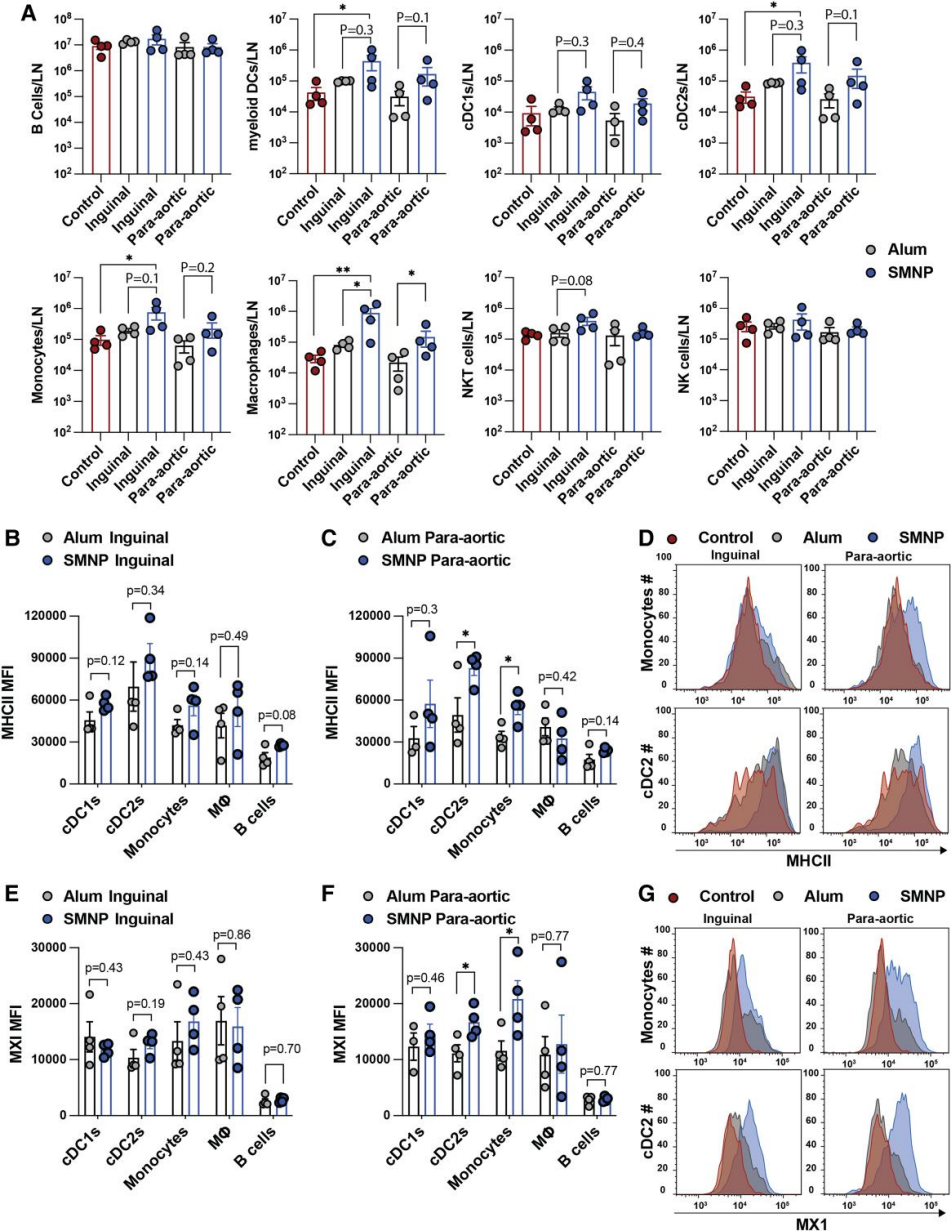
SMNP expands innate immune cell populations and triggers IFN signaling in both proximal and distal dLNs. LNs collected from NHPs following PET imaging at 48 h postimmunization with alum or SMNP adjuvant were processed for flow cytometry analysis. A) Cell counts from proximal inguinal and distal para-aortic LNs compared with control contralateral LNs. B–D) MHC II expression on APC populations. Shown are MFI values for inguinal LN (B), para-aortic LN (C), and representative histograms of MHC expression on monocytes (D). E–G) Intracellular straining for MX1 expression. Shown are MFI values for inguinal LN (E), para-aortic LN (F), and representative histograms of MX1 expression (G). Statistical comparisons were performed using one-way ANOVA, followed by Tukey's post hoc test (A) or Student's t test (B, C, E, F). *P < 0.05, **P < 0.01.

To verify conclusions suggested by the scRNA-seq analysis, we carried out flow cytometry analysis on proximal inguinal and distal para-aortic LNs recovered from NHPs post-PET imaging (Fig. S8). Profiling of innate and B cells revealed that SMNP induced large increases in the number of CD68^+^ macrophages and monocytes, as well as consistent trends toward increased numbers of CD123^+^ plasmacytoid and cDCs compared with alum in proximal draining inguinal LNs (Fig. 5A). Similar patterns of expanded innate immune cell populations in SMNP-vaccinated animals were also measured in more distal para-aortic LNs (Fig. 5A). We next examined markers of APC activation. In proximal inguinal LNs, MHC II expression trended higher on DCs, monocytes, macrophages, and B cells in SMNP-vaccinated nodes vs. alum-vaccinated nodes, but did not reach statistical significance for any of these populations (Fig. 5B). However, in more distal para-aortic LNs, MHC II was strongly up-regulated on cDC2 cells and monocytes in response to SMNP but not alum (Fig. 5C and D). We also examined CD80 expression on APCs, but no clear differences between groups were detected (Fig. S9). Finally, we carried out intracellular staining for expression of MX1, as an indicator of type I IFN signaling. We saw only a trend toward elevated MX1 expression in cDC2 and monocytes for in proximal inguinal nodes of SMNP-vaccinated animals (Fig. 5E), but at the more distal para-aortic LNs, MX1 expression was significantly up-regulated in these cell types (Fig. 5F and G). These data suggest that SMNP not only increases antigen delivery to more distal LNs, but also triggers innate immune stimulation necessary for promoting adaptive responses in these more distal sites.

## Discussion

Adjuvants are critical components of vaccines that enhance protective immune responses, especially against poorly immunogenic antigens. Saponin adjuvants formulated with lipids have been shown to be safe for human use and are used in both licensed vaccines and ongoing clinical trials (9, 44–46). SMNP is a recently developed ISCOM-type saponin adjuvant incorporating the TLR4 agonist MPLA, which has shown promising immunogenicity in diverse preclinical models (11, 47–50), and recently entered first-in-humans testing in a phase I clinical trial (HVTN144, clinicaltrials.gov: NCT06033209). Here, we performed a comparative analysis between SMNP and the traditional alum adjuvant in NHPs, to evaluate the efficacy of this adjuvant in eliciting immune responses, and to further understand its mechanisms of action. Injections were administered subcutaneously, a route historically validated in NHPs for generating strong immune responses (51–53). Inguinal LNs, which are readily accessed for longitudinal FNA sampling, are direct dLNs for subcutaneous vaccination in the thigh, which enabled our detailed tracking of ongoing GC responses in the animals over time. In addition, this injection site was selected to enable comparisons to the findings from our previous pilot study in RMs (11). SMNP for these studies was prepared using a highly purified saponin fraction termed QS-21, which is the form of saponin being used in the clinical trial of SMNP and is also the form of saponin employed in other saponin-based clinical adjuvant products such as GSK's AS01b and ALFQ developed by the US Army (9, 44). When evaluated with a native-like stabilized HIV Env trimer immunogen, we found that SMNP elicited much stronger immunity than the gold standard alum adjuvant Alhydrogel, with greatly expanded GC and follicular helper T-cell responses, elevated serum binding and neutralizing antibody titers, increased memory B-cell production, and increased BM plasma cell numbers. Mechanistically, we found through whole-animal and ex vivo PET/CT imaging that SMNP promoted antigen delivery to a greater pool of dLNs than alum, and induced a strong type I IFN signature associated with expanded innate immune cell populations and APC activation in both proximal and more distal dLNs.

One notable observation from the immunogenicity study was the significantly stronger antigen-specific GC response elicited by SMNP adjuvanted immunization, as determined by FNAs. GC B cells recognizing intact trimer expanded continuously over 8 WPPI, and were further sharply escalated following the first boost. This was particularly noteworthy given the established relationship between early GC responses and the induction of neutralizing responses to HIV trimers (54). These early GC responses and output tier 2 neutralizing antibody titers primed by SMNP were notably stronger than we observed in a parallel study comparing Quil-A-based SMNP with alum and 3M-052 adjuvant (47), which might reflect increased potency of the purified QS-21 saponin used here. GC responses were more muted after the second boost, likely due to the high levels of preexisting antibody titers by that time point.

The most striking observation made in these studies was the impact of SMNP adjuvant on the biodistribution of antigen following injection. In mice, we found that SMNP has multiple mechanisms of action that impact antigen trafficking, including induction of increased lymph flow, depletion of subcapsular sinus (SCS) macrophages, and augmented antigen entry in dLNs (11). These mechanisms correlated with the induction of strong GC responses in both immediate draining and more distal dLNs in mice, but the small distances between LNs in rodents made it unclear if these effects would be observed in a large animal model closer to humans. Using PET/CT imaging to visualize antigen biodistribution in NHPs, we found that SMNP did indeed promote antigen dissemination to a greater number of LNs in macaques, with antigen reaching up to 8 LNs in the draining lymphatic chain. In contrast, antigen release from the alum injection site was slower. However, despite ∼50% of the antigen being released from the alum injection site by 48 h postinjection, antigen levels in distal LNs (e.g. common iliac and paraaortic) still remained negligible. Efficient antigen accumulation in dLNs triggered by SMNP is likely facilitated not only by rapid clearance from the injection site but also by mechanisms, such as increased lymph flow and SCS macrophage depletion at the dLNs, we previously described in mice (11). Importantly, we found evidence that SMNP was also activating innate immune responses across the LN chain, as innate immune cell populations were expanded and enhanced APC activation relative to alum was detected in both proximal and more distal LNs. We attempted to detect the induction of B-cell priming across these dLNs, but at this very early time point of tissue sampling (48 h), no clear antigen-specific B-cell response could be measured.

In comparing SMNP with other saponin-based adjuvants like AS01b, our previous study in mice showed that SMNP elicited significantly higher antibody responses following the booster dose, along with enhanced Tfh and antigen-specific GC B-cell responses compared with AS01b. However, both SMNP and AS01b were found to deplete CD169^+^ macrophages in the SCS of dLNs, suggesting that they enhance antigen trafficking by altering LN structure. Additionally, ISCOM particles containing saponin but lacking the TLR4 agonist also enhanced antigen trafficking in mice, indicating that this may be a common feature of saponin-based adjuvants. Thus, our findings in NHPs may be broadly relevant to other clinical saponin adjuvants, such as AS01b and Matrix M (8–10).

NHPs are one of the closest-to-human animal models, especially in the pathophysiology of viral infections (55). However, aside from the broad atlases of tissues from wildtype *Macaca fascicularis* (56) and *Rhesus macaques* (57), to our knowledge, the impact of vaccine formulations on NHP LN myeloid cells at the single-cell transcriptomic level has not yet been examined. With scRNA-seq analysis, we found that even at the proximal LNs, SMNP induced a very distinct transcriptional program compared with alum. These findings echo transcriptional differences seen in an early microarray study profiling innate immune cells in the peripheral blood of NHPs vaccinated with eight different adjuvants combined with HIV Env antigen (58). Here, the authors found that TLR4-containing formulations induced inflammatory and myeloid-associated transcriptional modules while ISCOMs led to IFN and antiviral modules (58). Interestingly, SMNP, which is an ISCOM saponin particle and carries a TLR4 agonist, induced a response that appears to be a hybrid of these two signatures—eliciting monocyte, DC, and NKT cell expansion in the LNs and strong type I IFN responses across all major cell lineages (Fig. 4D and I). Type I IFNs are suggested to play a key role in promoting humoral immunity. They have been shown to enhance GC formation in mice (59), and patients with COVID-19 who have antibodies against type I IFNs exhibit reduced antibody production (60). Moreover, increased type I IFN signaling has been correlated with stronger B-cell responses to adjuvants like amphiphile-modified variants of CpG (61). These findings indicate that type I IFNs are likely key contributors to the strong B-cell responses observed with SMNPs.

In summary, our studies demonstrate a strong immunogenicity of QS-21-based SMNP adjuvant in NHPs, with induction of novel mechanisms of action in NHPs that, to our knowledge, have not been described for other vaccine adjuvants. These results provide further insight into the potency of SMNP across preclinical animal models and demonstrate some conservation of mechanisms of action between mice and larger animals. Importantly, SMNP was also found to be safe in NHPs, eliciting minimal reactogenicity or injection-site reactions, which will be a critical element for successful human use of this new adjuvant.

## Materials and methods

### Immunogen synthesis

The MD39 immunogen is a hyperstabilized BG505 SOSIP trimer and was synthesized, as described previously (19, 62). Briefly, trimer genes containing C-terminal His-tags were transfected into 293F cells grown in 293 Freestyle media (Life Technologies) by transient transfection with 293Fectin (Invitrogen). Protein was harvested from the culture supernatant at 5 days posttransfection and purified by nickel affinity chromatography on a His-Trap HP column (Cytiva) followed by size exclusion chromatography using a HiLoad 16/600 Superdex 200 column (Cytiva). The purity and molecular weight of the purified trimer were confirmed by reducing and nonreducing sodium dodecyl sulfate–polyacrylamide gel electrophoresis (SDS-PAGE).

### SMNP synthesis

SMNP is synthesized by self-assembly of dipalmitoylphosphatidyl choline (DPPC), MPLA, cholesterol, and saponin dissolved in MEGA-10 detergent at a 1:1:2:10 (DPPC:MPLA:Chol:Quil-A) mass ratio. Lipids, cholesterol, and synthetic MPLA (PHAD Phosphorylated Hexaacyl Disaccharide) were purchased from Avanti Lipids and used as received. QS-21 saponin was obtained from Desert King and used as received. SMNP was synthesized as previously described (11), replacing Quil-A saponin in the composition. The adjuvant was characterized by dynamic light scattering, high-performance liquid chromatography (HPLC), and a cholesterol quantification assay.

### Rhesus macaques

Female Indian RMs (*Macaca mulatta*) were used in this study, aged between 3 and 4 years at time of first immunization. They were housed at the New Iberia Primate Research Center and maintained in accordance with NIH guidelines. The research protocols for these studies were reviewed and approved by the Institutional Animal Care and Use Committee of the University of Louisiana at Lafayette (Protocol #2017-8789-005).

### Macaque immunization study and sample collection

Groups of six female RMs were immunized subcutaneously in the caudal thigh muscle at weeks 0, 8, 16, and 24 with 50 µg MD39 trimer formulated with 500 µg alum or 188 µg of SMNP, administered bilaterally in each thigh. For longitudinal titer studies, blood samples were collected by venipuncture from the femoral vein using serum collection tubes, frozen, and stored at −80 °C until analysis. Rectal and vaginal mucosal samples were collected using Merocel sponges, as previously described (63), and stored at −80 °C until analysis.

For PET imaging studies, MD39 trimer protein was labeled with DOTA-NHS-ester (Macrocyclics) in 0.1 M sodium phosphate buffer pH 7.3 previously treated with Chelex 100 chelating resin (Bio-Rad), and reacted at 4 °C for 18 h. Labeled trimer was purified using a Sephadex G-25 PD-10 Desalting column (GE) and stored at −80 °C until use. ^64^Cu was obtained from the WIMR Cyclotron Labs at the University of Madison, Wisconsin, and DOTA-labeled MD39 trimer was complexed with the radiometal in chelexed 0.1 M sodium acetate buffer (pH 5.5) at 37 °C with occasional mixing. Unbound ^64^Cu was removed from labeled MD39 by centrifugation through 10 kDa microfilters (Amicon Ultra, UFC 5010BK) at 10,000 × *g* for 10 min followed by two washes with pH 7.4 phosphate-buffered saline (PBS), and the radioactivity measured in a dose calibrator. Two groups of four female RMs each were immunized subcutaneously in two cohorts with 50 µg ^64^Cu-labeled MD39 adjuvanted with either alum or SMNP. In the first cohort of animals, macaques were injected with 2.1 mCi ^64^Cu; in the second cohort, animals received 3.8 mCi ^64^Cu. These animals were then subjected to PET/CT scanning at 2, 4, 6, 12, 24, and 48 h postvaccine administration.

### Enzyme-linked immunosorbent assay measurement of antibody titers

Antitrimer titer was measured by enzyme-linked immunosorbent assay (ELISA). Nunc MaxiSorp plates were coated streptavidin overnight at 4 °C and blocked with 2% bovine serum albumin in PBS. Blocked plates were washed with washing buffer (PBS with 0.05% Tween-20) and incubated with Avi-tagged biotinylated antigen overnight at 4 °C. Plates were washed again, and serial dilutions of sera were added to plates and incubated for 2 h at room temperature, following which the plates were washed and incubated with antirhesus IgG-HRP (and its subclasses), IgA-HRP, and IgM-HRP (Bio-Rad). ELISA plates were developed with TMB substrate and stopped by sulfuric acid 2M. Absorbance values at 450 nm were measured, with a background correction wavelength of 540 nm. Serum titer was defined as the logarithm of the reciprocal of serum dilution that resulted in absorbance readings of >0.1 unit above background.

### PET/CT imaging and analysis

All whole-body PET/CT scans were performed on a Philips Gemini TF64 scanner. Acquisitions were done in 3D mode with an axial field of view of 57.6 cm. The PET and CT data were exported as DICOM files and analyzed using the Multi-Image Analysis GUI (MANGO, Research Imaging Institute, UT Health San Antonio). Decay-adjusted PET images (corrected to the time of vaccine injection) were normalized by the injected doses and body weight to generate SUV maps. To designate regions of interest (ROI), spherical outlines were placed on target tissues, freehand outline drawings were made, and/or tissue contours on the SUV maps were identified through signal thresholding. These ROIs were then subjected to statistical evaluations to determine SUVmax, SUVmean, and SUVsum. The 3D image data were exhibited as color-coded maximum intensity projections of the SUVmax maps. Analysis of the distal LNs was performed by drawing 3D ROIs for the injection site and the most distal LN with a significant detectable signal (>10% of the maximum signal); these were subsequently projected onto a 3D surface-rendered PET image. The vaccine’s travel distance was measured by drawing a straight line on the 3D image between the SUVmax points of the injection site and the relevant LN.

Following the 48-h time point PET imaging session, the animals were euthanized in a humane manner with an intravenous administration of 200 mg/kg Beuthanasia-D. An experienced veterinary pathologist then performed necropsies, and selected LNs underwent secondary PET imaging using the protocol described above.

## Supplementary Material

pgae529_Supplementary_Data

## Data Availability

Single-cell RNA-seq data, including raw sequencing reads, processed count matrices, and annotated Seurat objects, have been deposited at GEO with the accession number GSE264685 and the reviewer access number kzorcyewbrefril.

## Preprints

This manuscript was posted on a preprint: https://www.biorxiv.org/content/10.1101/2024.08.28.608716v1.

## References

[1] Pulendran B, Arunachalam PS, O’Hagan DT. 2021. Emerging concepts in the science of vaccine adjuvants. Nat Rev Drug Discov. 20:454–475. 33824489 10.1038/s41573-021-00163-y PMC8023785

[2] Giudice GD, Rappuoli R, Didierlaurent AM. 2018. Correlates of adjuvanticity: a review on adjuvants in licensed vaccines. Semin Immunol. 39:14–21. 29801750 10.1016/j.smim.2018.05.001

[3] Mertz TM, Collins CD, Dennis M, Coxon M, Roberts SA. 2022. APOBEC-induced mutagenesis in cancer. Annu Rev Genet. 56:229–252. 36028227 10.1146/annurev-genet-072920-035840

[4] Ratnapriya S, Perez-Greene E, Schifanella L, Herschhorn A. 2022. Adjuvant-mediated enhancement of the immune response to HIV vaccines. FEBS J. 289:3317–3334. 33705608 10.1111/febs.15814

[5] Wei C-J, et al 2020. Next-generation influenza vaccines: opportunities and challenges. Nat Rev Drug Discov. 19:239–252. 32060419 10.1038/s41573-019-0056-x PMC7223957

[6] Su S, Li W, Jiang S. 2022. Developing pan-β-coronavirus vaccines against emerging SARS-CoV-2 variants of concern. Trends Immunol. 43:170–172. 35125310 10.1016/j.it.2022.01.009 PMC8758279

[7] de Groot C, Müller-Goymann C. 2016. Saponin interactions with model membrane systems—Langmuir monolayer studies, hemolysis and formation of ISCOMs. Planta Med. 82:1496–1512. 27760443 10.1055/s-0042-118387

[8] Didierlaurent AM, et al 2017. Adjuvant system AS01: helping to overcome the challenges of modern vaccines. Expert Rev Vaccines. 16:55–63. 27448771 10.1080/14760584.2016.1213632

[9] Didierlaurent AM, et al 2017. Chapter 14—the development of the adjuvant system AS01: a combination of two immunostimulants MPL and QS-21 in liposomes. In: Schijns VEJC, O’Hagan DT, editors. Immunopotentiators in modern vaccines. Academic Press. p. 265–285.

[10] Shinde V, et al 2021. Efficacy of NVX-CoV2373 COVID-19 vaccine against the B.1.351 variant. N Engl J Med. 384:1899–1909. 33951374 10.1056/NEJMoa2103055 PMC8091623

[11] Silva M, et al 2021. A particulate saponin/TLR agonist vaccine adjuvant alters lymph flow and modulates adaptive immunity. Sci Immunol. 6:eabf1152. 34860581 10.1126/sciimmunol.abf1152 PMC8763571

[12] Pifferi C, Fuentes R, Fernández-Tejada A. 2021. Natural and synthetic carbohydrate-based vaccine adjuvants and their mechanisms of action. Nat Rev Chem. 5:197–216. 33521324 10.1038/s41570-020-00244-3 PMC7829660

[13] Marty-Roix R, et al 2016. Identification of QS-21 as an inflammasome-activating molecular component of saponin adjuvants. J Biol Chem. 291:1123–1136. 26555265 10.1074/jbc.M115.683011 PMC4714196

[14] den Brok MH, et al 2016. Saponin-based adjuvants induce cross-presentation in dendritic cells by intracellular lipid body formation. Nat Commun. 7:13324. 27819292 10.1038/ncomms13324 PMC5103066

[15] Wilson NS, et al 2014. Inflammasome-dependent and -independent IL-18 production mediates immunity to the ISCOMATRIX adjuvant. J Immunol. 192:3259–3268. 24610009 10.4049/jimmunol.1302011

[16] Wilson NS, et al 2012. ISCOMATRIX vaccines mediate CD8+ T-cell cross-priming by a MyD88-dependent signaling pathway. Immunol Cell Biol. 90:540–552. 21894173 10.1038/icb.2011.71 PMC3365289

[17] Schnurr M, et al 2009. ISCOMATRIX adjuvant induces efficient cross-presentation of tumor antigen by dendritic cells via rapid cytosolic antigen delivery and processing via tripeptidyl peptidase II. J Immunol. 182:1253–1259. 19155470 10.4049/jimmunol.182.3.1253

[18] Morelli AB, et al 2012. ISCOMATRIX: a novel adjuvant for use in prophylactic and therapeutic vaccines against infectious diseases. J Méd Microbiol. 61:935–943. 22442293 10.1099/jmm.0.040857-0

[19] Steichen JM, et al 2016. HIV vaccine design to target germline precursors of glycan-dependent broadly neutralizing antibodies. Immunity. 45:483–496. 27617678 10.1016/j.immuni.2016.08.016 PMC5040827

[20] Havenar-Daughton C, et al 2016. Direct probing of germinal center responses reveals immunological features and bottlenecks for neutralizing antibody responses to HIV Env trimer. Cell Rep. 17:2195–2209. 27880897 10.1016/j.celrep.2016.10.085 PMC5142765

[21] Burny W, et al 2017. Different adjuvants induce common innate pathways that are associated with enhanced adaptive responses against a model antigen in humans. Front Immunol. 8:943. 28855902 10.3389/fimmu.2017.00943 PMC5557780

[22] Burny W, et al 2019. Inflammatory parameters associated with systemic reactogenicity following vaccination with adjuvanted hepatitis B vaccines in humans. Vaccine. 37:2004–2015. 30850240 10.1016/j.vaccine.2019.02.015

[23] Steensberg A, Fischer CP, Keller C, Møller K, Pedersen BK. 2003. IL-6 enhances plasma IL-1ra, IL-10, and cortisol in humans. Am J Physiol Endocrinol Metab. 285:E433–E437. 12857678 10.1152/ajpendo.00074.2003

[24] Chang WLW, et al 2017. Changes in circulating B cell subsets associated with aging and acute SIV infection in rhesus macaques. PLoS One. 12:e0170154. 28095513 10.1371/journal.pone.0170154 PMC5240950

[25] Li J, et al 2023. Mature dendritic cells enriched in immunoregulatory molecules (mregDCs): a novel population in the tumour microenvironment and immunotherapy target. Clin Transl Med. 13:e1199. 36808888 10.1002/ctm2.1199 PMC9937888

[26] Collin M, Bigley V. 2018. Human dendritic cell subsets: an update. Immunology. 154:3–20. 29313948 10.1111/imm.12888 PMC5904714

[27] Eisenbarth SC . 2019. Dendritic cell subsets in T cell programming: location dictates function. Nat Rev Immunol. 19:89–103. 30464294 10.1038/s41577-018-0088-1 PMC7755085

[28] Brown CC, et al 2019. Transcriptional basis of mouse and human dendritic cell heterogeneity. Cell. 179:846–863.e24. 31668803 10.1016/j.cell.2019.09.035 PMC6838684

[29] Sander J, et al 2017. Cellular differentiation of human monocytes is regulated by time-dependent interleukin-4 signaling and the transcriptional regulator NCOR2. Immunity. 47:1051–1066.e12. 29262348 10.1016/j.immuni.2017.11.024 PMC5772172

[30] Wu X, et al 2016. Transcription factor Zeb2 regulates commitment to plasmacytoid dendritic cell and monocyte fate. Proc Natl Acad Sci U S A. 113:14775–14780. 27930303 10.1073/pnas.1611408114 PMC5187668

[31] Staiano RI, et al 2009. Expression and function of Na^+^/Ca^2+^ exchangers 1 and 3 in human macrophages and monocytes. Eur J Immunol. 39:1405–1418. 19350557 10.1002/eji.200838792

[32] Alcántara-Hernández M, et al 2017. High-dimensional phenotypic mapping of human dendritic cells reveals interindividual variation and tissue specialization. Immunity. 47:1037–1050.e6. 29221729 10.1016/j.immuni.2017.11.001 PMC5738280

[33] Villani A-C, et al 2017. Single-cell RNA-seq reveals new types of human blood dendritic cells, monocytes, and progenitors. Science. 356:eaah4573. 10.1126/science.aah4573 PMC5775029 28428369

[34] Leylek R, et al 2019. Integrated cross-species analysis identifies a conserved transitional dendritic cell population. Cell Rep. 29:3736–3750.e8. 31825848 10.1016/j.celrep.2019.11.042 PMC6951814

[35] Nutt SL, Chopin M. 2020. Transcriptional networks driving dendritic cell differentiation and function. Immunity. 52:942–956. 32553180 10.1016/j.immuni.2020.05.005

[36] Sulczewski FB, et al 2023. Transitional dendritic cells are distinct from conventional DC2 precursors and mediate proinflammatory antiviral responses. Nat Immunol. 24:1265–1280. 37414907 10.1038/s41590-023-01545-7 PMC10683792

[37] Schneider WM, Chevillotte MD, Rice CM. 2014. Interferon-stimulated genes: a complex web of host defenses. Annu Rev Immunol. 32:513–545. 24555472 10.1146/annurev-immunol-032713-120231 PMC4313732

[38] Cui A, et al 2024. Dictionary of immune responses to cytokines at single-cell resolution. Nature. 625:377–384. 38057668 10.1038/s41586-023-06816-9 PMC10781646

[39] Liberzon A, et al 2015. The molecular signatures database hallmark gene set collection. Cell Syst. 1:417–425. 26771021 10.1016/j.cels.2015.12.004 PMC4707969

[40] Blanco P, Palucka AK, Gill M, Pascual V, Banchereau J. 2001. Induction of dendritic cell differentiation by IFN-α in systemic lupus erythematosus. Science. 294:1540–1543. 11711679 10.1126/science.1064890

[41] Cucak H, Yrlid U, Reizis B, Kalinke U, Johansson-Lindbom B. 2009. Type I interferon signaling in dendritic cells stimulates the development of lymph-node-resident T follicular helper cells. Immunity. 31:491–501. 19733096 10.1016/j.immuni.2009.07.005

[42] Jego G, et al 2003. Plasmacytoid dendritic cells induce plasma cell differentiation through type I interferon and interleukin 6. Immunity. 19:225–234. 12932356 10.1016/s1074-7613(03)00208-5

[43] Banchereau J, Pascual V. 2006. Type I interferon in systemic lupus erythematosus and other autoimmune diseases. Immunity. 25:383–392. 16979570 10.1016/j.immuni.2006.08.010

[44] Alving CR, Peachman KK, Matyas GR, Rao M, Beck Z. 2020. Army liposome formulation (ALF) family of vaccine adjuvants. Expert Rev Vaccines. 19:279–292. 32228108 10.1080/14760584.2020.1745636 PMC7412170

[45] Lenart K, et al 2024. Three immunizations with Novavax's protein vaccines increase antibody breadth and provide durable protection from SARS-CoV-2. npj Vaccines. 9:17. 38245545 10.1038/s41541-024-00806-2 PMC10799869

[46] Heath PT, et al 2021. Safety and efficacy of NVX-CoV2373 COVID-19 vaccine. N Engl J Med. 385:1172–1183. 34192426 10.1056/NEJMoa2107659 PMC8262625

[47] Phung I, et al 2023. A combined adjuvant approach primes robust germinal center responses and humoral immunity in non-human primates. Nat Commun. 14:7107. 37925510 10.1038/s41467-023-42923-x PMC10625619

[48] Hartwell BL, et al 2022. Intranasal vaccination with lipid-conjugated immunogens promotes antigen transmucosal uptake to drive mucosal and systemic immunity. Sci Transl Med. 14:eabn1413. 10.1126/scitranslmed.abn1413 PMC9835395 35857825

[49] Lee JH, et al 2022. Long-primed germinal centres with enduring affinity maturation and clonal migration. Nature. 609:998–1004. 36131022 10.1038/s41586-022-05216-9 PMC9491273

[50] Rodrigues KA, et al 2021. Phosphate-mediated coanchoring of RBD immunogens and molecular adjuvants to alum potentiates humoral immunity against SARS-CoV-2. Sci Adv. 7:eabj6538. 10.1126/sciadv.abj6538 PMC8654298 34878851

[51] Lampe K, et al 2017. Immunization of rhesus macaques with *Echinococcus multilocularis* recombinant 14-3-3 antigen leads to specific antibody response. Parasitol Res. 116:435–439. 27787625 10.1007/s00436-016-5303-z PMC5167771

[52] Ols S, et al 2020. Route of vaccine administration alters antigen trafficking but not innate or adaptive immunity. Cell Rep. 30:3964–3971.e7. 32209459 10.1016/j.celrep.2020.02.111 PMC7198771

[53] Schiffner T, et al 2024. Vaccination induces broadly neutralizing antibody precursors to HIV gp41. Nat Immunol. 25:1073–1082. 38816615 10.1038/s41590-024-01833-w PMC11147780

[54] Pauthner M, et al 2017. Elicitation of Robust Tier 2 neutralizing antibody responses in nonhuman primates by HIV envelope trimer immunization using optimized approaches. Immunity. 46:1073–1088.e6. 28636956 10.1016/j.immuni.2017.05.007 PMC5483234

[55] Estes JD, Wong SW, Brenchley JM. 2018. Nonhuman primate models of human viral infections. Nat Rev Immunol. 18:390–404. 29556017 10.1038/s41577-018-0005-7 PMC5970954

[56] Han L, et al 2022. Cell transcriptomic atlas of the non-human primate *Macaca fascicularis*. Nature. 604:723–731. 35418686 10.1038/s41586-022-04587-3

[57] Staupe RP, et al 2022. Single cell multi-omic reference atlases of non-human primate immune tissues reveals CD102 as a biomarker for long-lived plasma cells. Commun Biol. 5:1399. 36543997 10.1038/s42003-022-04216-9 PMC9770566

[58] Francica JR, et al 2017. Innate transcriptional effects by adjuvants on the magnitude, quality, and durability of HIV envelope responses in NHPs. Blood Adv. 1:2329–2342. 29296883 10.1182/bloodadvances.2017011411 PMC5729628

[59] Dahlgren MW, et al 2022. Type I interferons promote germinal centers through B cell intrinsic signaling and dendritic cell dependent Th1 and Tfh cell lineages. Front Immunol. 13:932388. 35911733 10.3389/fimmu.2022.932388 PMC9326081

[60] Aoki A, et al 2024. Suppression of type I interferon signaling in myeloid cells by autoantibodies in severe COVID-19 patients. J Clin Immunol. 44:104. 38647550 10.1007/s10875-024-01708-7 PMC11035476

[61] Seenappa LM, et al 2022. Amphiphile-CpG vaccination induces potent lymph node activation and COVID-19 immunity in mice and non-human primates. npj Vaccines. 7:128. 36307453 10.1038/s41541-022-00560-3 PMC9616425

[62] Martin JT, et al 2021. Combined PET and whole-tissue imaging of lymphatic-targeting vaccines in non-human primates. Biomaterials. 275:120868. 34091299 10.1016/j.biomaterials.2021.120868 PMC8325633

[63] Kozlowski PA, et al 2000. Modified wick method using Weck-Cel sponges for collection of human rectal secretions and analysis of mucosal HIV antibody. J Acquir Immune Defic Syndr. 4:297–309. 10.1097/00126334-200008010-00001 11015145

