# Supplementary material for: Modulation of antigen delivery and lymph node activation in nonhuman primates by saponin adjuvant saponin/monophosphoryl lipid A nanoparticle
Source: PNAS Nexus. 2024 Nov 25;3(12):pgae529. doi: 10.1093/pnasnexus/pgae529 (PMC11645456; doi:10.1093/pnasnexus/pgae529)
Supplement: pgae529_Supplementary_Data [file pgae529_supplementary_data.pdf]

SUPPLEMENTARY MATERIALS AND METHODS

Antigen-tetramer for trimer-specific B cell identification

To prepare MD39 probes, biotinylated MD39 trimer (prepared by biotinylating Avi-tagged MD39 protein using the Avidity LLC BirA kit according to the manufacturer's instructions) was mixed with either streptavidin-APC or streptavidin-BV605 in a 5:1 molar ratio. This mixture was then incubated for 20 minutes at room temperature to ensure proper tetramer formation before proceeding with the staining process.

Flow cytometry analysis of germinal center responses

To longitudinally track the germinal center responses, lymph node fine needle aspirates (FNAs) were used to sample both right and left inguinal LNs identified to be the primary draining LNs by palpation. FNAs were conducted by a veterinarian. Samples were collected by inserting a 22-gauge needle attached to a 3mL syringe into the lymph nodes 4 times, and dispensed into the complete RPMI media (RPMI containing 10% fetal bovine serum (FBS), and 1 μg/ml penicillin/streptomycin), and the needle was then flushed with media up to 3 times to collect all cells. Samples were centrifuged, and ACK (ammonium-chloride-potassium) lysis buffer was added if the sample had red blood cells. Cells were counted and cryopreserved in freezing medium (90% FBS, 10% DMSO) and stored in liquid nitrogen. For analysis, cells were thawed and dispersed through 70-μm cell strainers using the back of a 1-ml syringe plunger, and washed with PBS. The resulting single cell suspension was stained with Live/Dead Zombie Aqua for 15 minutes at room temperature, washed and then treated with antigen-tetramers for 30 minutes at room temperature. After pre-treatment with anti-CD16/32 Trustain FcX (10 minute at 4 °C) to block any nonspecific binding, cells were stained for 30 minutes at 4°C with antibodies (from BioLegend or BD Biosciences) against CD3, CD4, CD8, CD20, BCl-6, K67, PD1, and CXCR5. Antigen-specific staining was done using biotinylated trimer conjugated to streptavidin-BV421 (BioLegend) and streptavidin-APC (BioLegend). Antigen-specific GC B cells were gated as CD20+, CD3-, BCl-6+, Ki67+, MD39+ cells, and Tfh cells were gated as CD3+, CD20-, CD4+, CXCR5+, PD1+ cells.

Flow cytometry analysis of memory B cell responses

Frozen PBMC samples were thawed and recovered in RPMI media with 10% FBS, supplemented with 1X penicillin/streptomycin. Fluorescent antigen probes were generated by mixing small incremental volumes of fluorophore-conjugated streptavidin (streptavidin-BV421 and streptavidin-APC) with biotinylated antigen in 1x PBS at 25°C over 45 min. Zombie UV fixable viability dye (BioLegend) was used according to manufacturer's protocol. Cells were washed and incubated with Human TruStain FcX (Fc Receptor Blocking Solution) for 15 minutes at 25°C, followed by incubation with antigen probes for 30 minutes at 4 °C and then with surface antibodies for an additional 30 minutes at 4 °C. The staining antibody cocktail included: anti-human CD27 BUV395 (O323, BD Biosciences,1:20), CD20 BUV737 (L27, BD Biosciences, 1:20 dilution), IgM FITC (G20-127, BD, 1:20), IgD PE (Southern Biotech, 1:50), CD21 PE-Cy7 (B-ly4, BD, 1:20), CD14 APC-Cy7 (M5E2, BioLegend,

1:20), CD16 eFluor780 (3G8, Invitrogen, 1:20), CD3 APC-Cy7 (SP34-2, BD Biosciences, 1:20). After staining cells were fixed with 4% paraformaldehyde for 20 minutes at 4°C. Samples were spiked with Precision Count Beads and cell numbers were calculated according to the manufacturer's protocol. At least 500,000 events per sample were acquired on an FACSymphony A3 (BD Biosciences) and data was analyzed using FlowJo v10 (FlowJo Inc).

Bone marrow ELISPOT

Antibody-secreting cells (ASCs) in the bone marrow (BM) were assessed by enzyme-linked immune absorbent spot (ELISpot) assay. Briefly, 96-well multiscreen HTS filter plates (MilliporeSigma, catalog no. MSHAN4B50) were coated overnight at 4°C with 10 µg/ml of anti-monkey IgG, or IgM goat antibody (Rockland) or with 2 µg/ml of MD39 trimer for total or antigen-specific ASCs in BM, respectively. The plates were washed 4 times with PBS-0.05% Tween 20 (PBS-T) and 4 times with PBS and blocked with complete RPMI media for 2 h in a 5% CO2 incubator at 37°C. The BM cells were collected from the iliac crest of the animal in EDTA and separated by ficoll gradients method. Cells were counted and resuspended in complete RPMI media as 1 million per ml for total, and 5 million per ml for antigen-specific, plated in serial 3-fold dilutions, respectively, and incubated overnight in a 5% CO2 incubator at 37°C. Plates were then washed 4 times with PBS-T, and incubated with 1:1,000 diluted (PBS-T with 1% FBS) of anti-monkey IgG-, or IgM-biotin-conjugated antibodies (Rockland), respectively, for 2 h at room temperature. The plates were again washed 4 times with PBS-T followed by addition of avidin D-horseradish peroxidase (HRP) (Vector Laboratories) diluted 1:1,000 in PBS-T with 1% FBS, for 1 h at room temperature. After washing 4 times with PBS-T and 4 times with PBS, plates were developed using the AEC substrate kit (BD Biosciences). To stop the reaction, plates were washed extensively with water followed by air drying. Spots were imaged and counted using the Immunospot ELISPOT Analyzer (Cellular Technology Limited). The number of spots specific for each Ig isotype was reported as the number of either total or antigen-specific ACSs per million BM cells.

Flow cytometry for innate cells and B cells

Lymph nodes of vaccinated macaques were isolated 48 hours post-immunization, at the completion of PET imaging. The collected lymph nodes were first scanned before being mechanically dissociated into single cell suspensions. Cells were filtered, counted, and resuspended in freezing medium before storage in liquid nitrogen for subsequent analysis by flow cytometry and single cell RNA sequencing.

For flow cytometry analysis, Zombie UV fixable viability dye (BioLegend) was used according to manufacturer's protocol. Cells were washed and incubated with Human TruStain FcX (Fc Receptor Blocking Solution) for 15 minutes at 25°C, followed by staining with a cocktail of fluorescent antibodies for 30 minutes at 4°C (panels 1-3 described below). After staining cells were either fixed with 4% paraformaldehyde for 20 minutes at 4°C (panel #2 and #3) or processed for intracellular staining with BD Cytofix/Cytoperm Fixation/Permeabilization Kit, according to the manufacturer's instructions (panel #1). Intracellular staining was carried out in BD Perm/Wash buffer for 30 minutes at 4°C. After staining, samples were spiked with Precision

Count Beads and cell numbers were calculated according to the manufacturer's protocol. At least 500,000 events per sample were acquired on an FACSymphony A3 (BD Biosciences) and data was analyzed using FlowJo v10 (FlowJo Inc).

Panel #1 included anti-human CD16 BUV396 (3G8, BDBiosciences, 1:20), CD14 BUV737 (M5E2, BD Biosciences, 1:20), CLEC9A BV421 (3A4, BD Biosciences, 1:10), CD80 BV650 (L307.4, BD Biosciences, 1:20), HLA-DR PE (Tu36, BioLegend, 1:20), CD11c PE-Cy7 (3.9, BioLegend, 1:20), CD68 AF467 (eBioY1/82A, ebioscience, 1:10), CD20 APC-Cy7 (L27, BD Biosciences, 1:20). Intracellular staining: anti-human MX1 CoraLite® Plus 488 (Invitrogen, 1:20).
Panel #2 included anti-human CD16 BUV396 (3G8, BD Biosciences, 1:20), CD123 BV421 (6H6, BioLegend, 1:20), CD80 BV650 (L307.4, BD Biosciences, 1:20), CD14 AF488 (M5E2, BioLegend, 1:20), HLA-DR PE (Tu36, BioLegend, 1:20), CD11c PE-Cy7 (3.9, BioLegend, 1:20), CD66abce APC (TET2, Miltenyi, 1:20), and CD20 APC-Cy7 (L27, BD Biosciences, 1:20). Panel #3 included anti-human CD8 BUV396 (RPA-T8, BD Biosciences, 1:20), CD20 BUV737 (L27, BD Biosciences, 1:20), CD80 BV650 (L307.4, BD Biosciences, 1:20), NKG2A-FITC (REA110, Miltenyi, 1:20), CD14 PerCP/Cy5.5 (M5E2, BioLegend, 1:20), HLA-DR PE (Tu36, BioLegend, 1:20), CD3 APC-Cy7 (SP34-2, BD Biosciences, 1:20).

Single-cell RNA sequencing preparation and analysis

Proximal inguinal LNs were collected from NHPs at 48 hours post immunization and processed into single cell suspensions as described above. Cells were stained for viability using Zombie NIR and with antibodies against CD3 (APC-Cy7, SP34-2), CD8 (APC-Cy7, RPA-T8), CD20 (APC-Cy7, L27), HLA-DR (PE, Tu36). Live $CD3^{low}CD8^{low}CD20^{low}$ cells were sorted on a BD FACS Aria (BD Biosciences) cell sorter, processed immediately using the commercial 5` Single Cell GEX v2 platform (10x Genomics) following the manufacturer's protocols, and sequenced on a NovaSeq 6000 system (Illumina).

Sequenced FASTQ files were aligned to the Rhesus macaques Mmul_10 genome assembly (Ensembl.org) using the cellranger cloud analysis pipeline (10x Genomics, v7.1.0) with default settings. Downstream analysis was performed in R (v4.3.2). The gene expression count matrices were processed and analyzed using Seurat (v5.0.1) (1). The initial quality control filtered out genes that were detected in less than 3 cells and removed cells with less than 500 genes, greater than 30000 UMI counts, and greater than 10% mitochondrial genes. Cells were log-normalized using the NormalizeData function, and variable genes were identified using the FindVariableFeatures function. The ScaleData function was used to scale the data to unit variance using a Poisson model and regress out RNA feature counts and percent of mitochondrial genes before performing principal component analysis (PCA) using the RunPCA function. Data integration was performed using the IntegrateLayers() function with the Harmony algorithm (v1.2.0) (2). Thirty principal components (PCs) were used for constructing the nearest-neighbor graph with the FindNeighbors function. Thirty neighboring points and thirty PCs were used to generate uniform manifold approximation and projection (UMAP) with the RunUMAP function. Unsupervised clustering was determined using Louvain clustering as implemented in the FindClusters function. Differential gene expression analysis was performed using FindMarkers function. Clusters with strong

expressions of marker genes associated with more than one cell population were denoted as doublet clusters and discarded from downstream analysis. The Hallmark interferon alpha response gene set was procured from the human Molecular Signatures Database (MSigDB), and the AddModuleScore function from Seurat was used to calculate module scores of the gene set for each cell.

Immune response enrichment analysis (IREA) was performed as described.(39) Briefly, a list of statistically upregulated genes (adjusted p-value < 0.01) in SMNP macrophages compared to alum macrophages was identified using the FindMarkers function in Seurat. This gene list was mapped onto a published scRNA-seq dataset with annotated macrophages treated with 66 different cytokines and PBS (39) to characterize how SMNP polarized macrophages. For each comparison, two sets of scores were calculated by summing the normalized expression values of genes in the SMNP gene list in each of the cytokine-treated macrophages and PBS-treated macrophages, and two-sided Wilcoxon rank-sum tests between the two sets of scores were used to assess statistical significance. An FDR correction is applied to all cytokine calculations.

Statistics

All graphs were prepared in GraphPad Prism Version 10.2.2 and statistical testing was performed using Prism. In the bar graphs, each symbol represents an individual monkey. Datasets were tested for normality and datasets showing lognormal distributions were transformed to linear scale before performing statistical analysis. Statistical comparisons were performed by Student's *t* test or one-way ANOVA followed by a post hoc test as specified in figure legends. Statistical significance was determined at levels of *p < 0.05, **p < 0.01, and ***p < 0.001.

References:

1. **Hao Y**, et al., Dictionary learning for integrative, multimodal and scalable single-cell analysis. *Nat. Biotechnol*. 42, 293–304 (2024).

2. **Korsunsky I**, et al., Fast, sensitive and accurate integration of single-cell data with Harmony. *Nat. Methods* 16, 1289–1296 (2019).

SUPPLEMEMTARY FIGURES

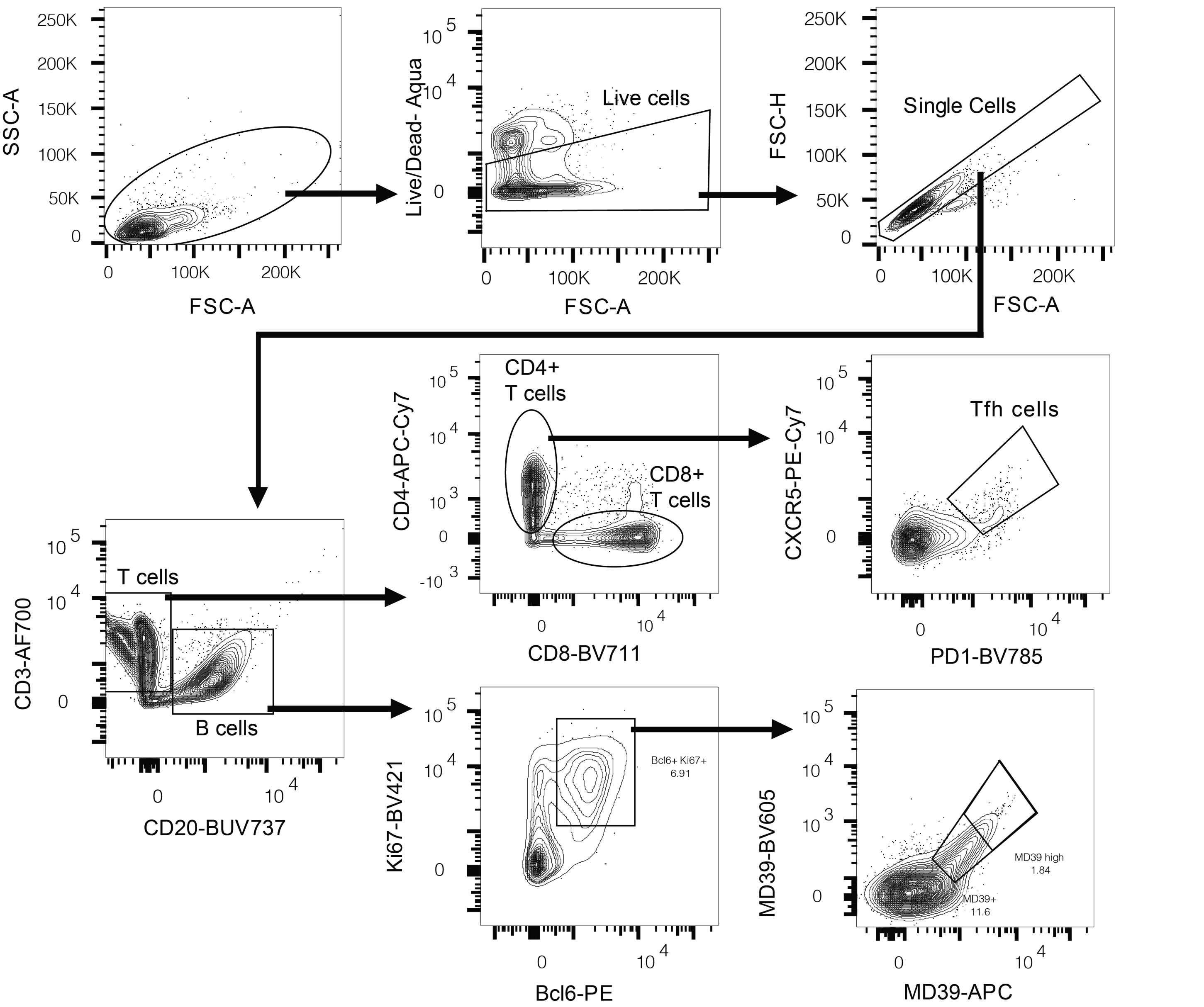

Figure S1: Flow cytometry gating strategy for analysis of GC Tfh and antigen-specific B cells. Lymphocytes isolated from FNAs were immunolabeled for the markers indicated. Live cells (negative for Zombie Aqua), pre-gated on FSC-A/FSC-H to identify singlets, were gated on CD3 and CD20 markers. B cells (CD3- CD20+) were gated on Ki67 and BCl-6 high expression to identify GC B cells and were then gated twice on MD39-tetramers for antigen-specificity. T cells (CD3+ CD20-) were gated PD1 and CXCR5 high expression to identify Tfh cells.

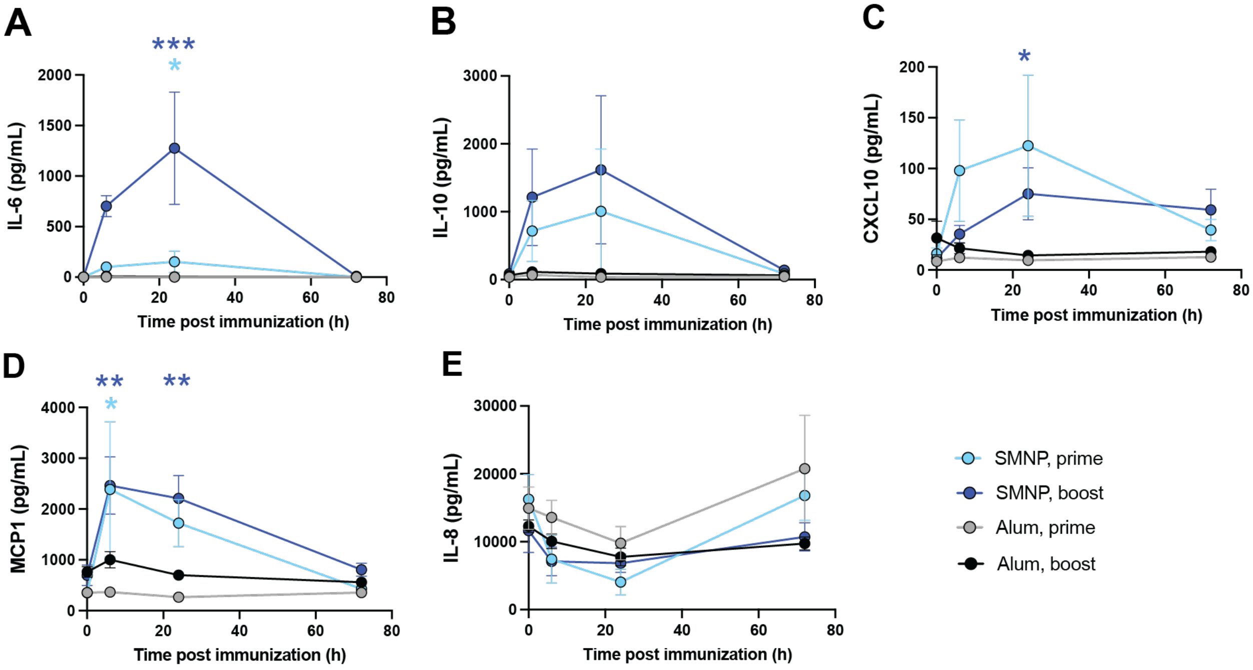

Figure S2: Cytokine responses to vaccination. The concentration of cytokines and chemokines in serum was measured at 6, 24, and 72 hours after the prime and first boost immunization. Cytokines analyzed included IL-6 (A), IL-10 (B), CXCL10 (C), MCP1 (D), and IL-8 (E). Statistical analyses were performed using one-way ANOVA, followed by Sidak's post-hoc test. (*P < 0.05, **P < 0.01, ***P < 0.001).

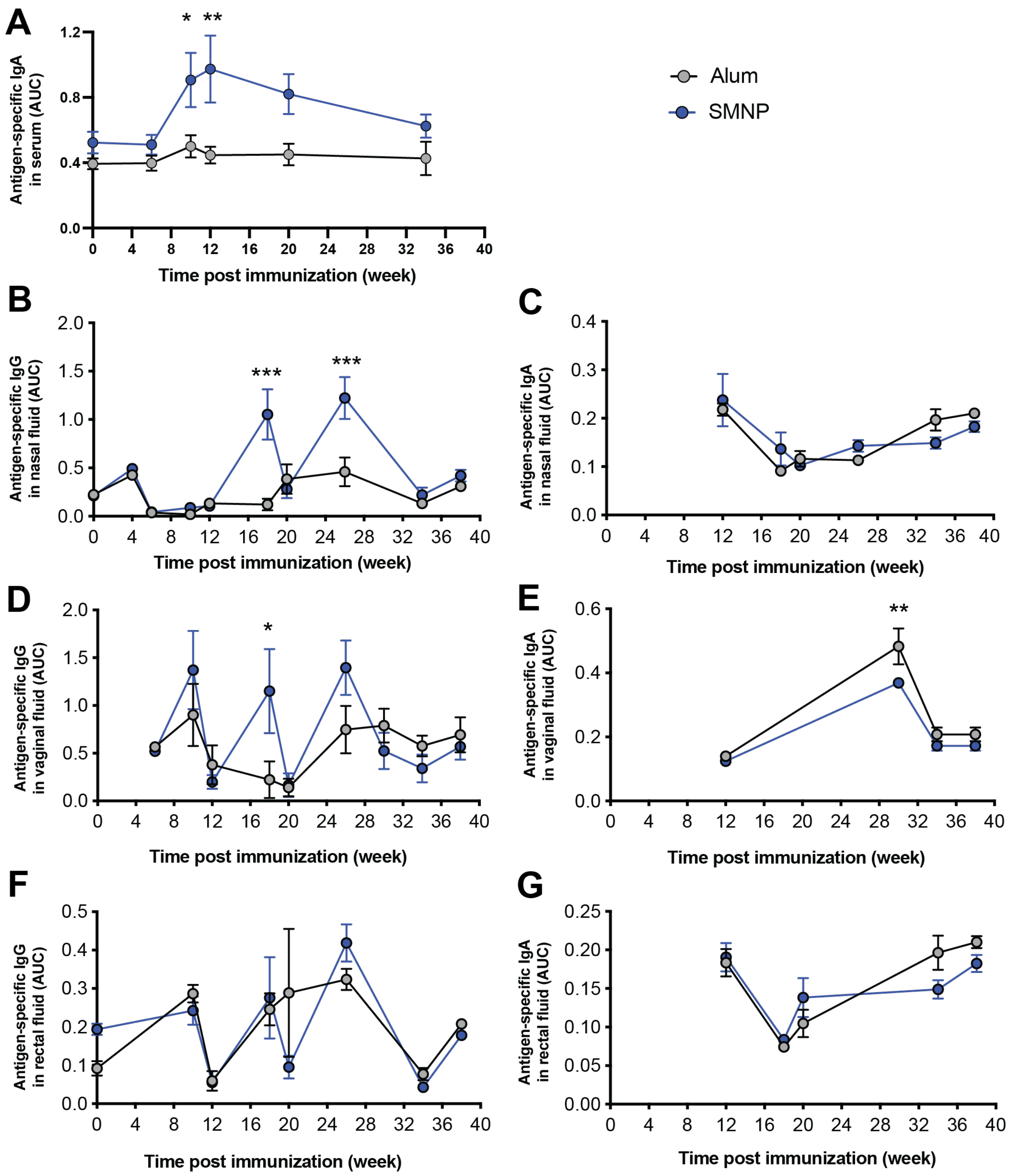

Figure S3: Systemic and mucosal antibody responses to vaccination. IgA and IgG were measured in serum and mucosal washes from the (A) nose, (B), (C) vagina and (D) the rectum. Data are presented as mean ± SEM. Statistical comparison between the two groups at each time point was performed by one-way ANOVA followed by Sidak's m post-hoc test was used.

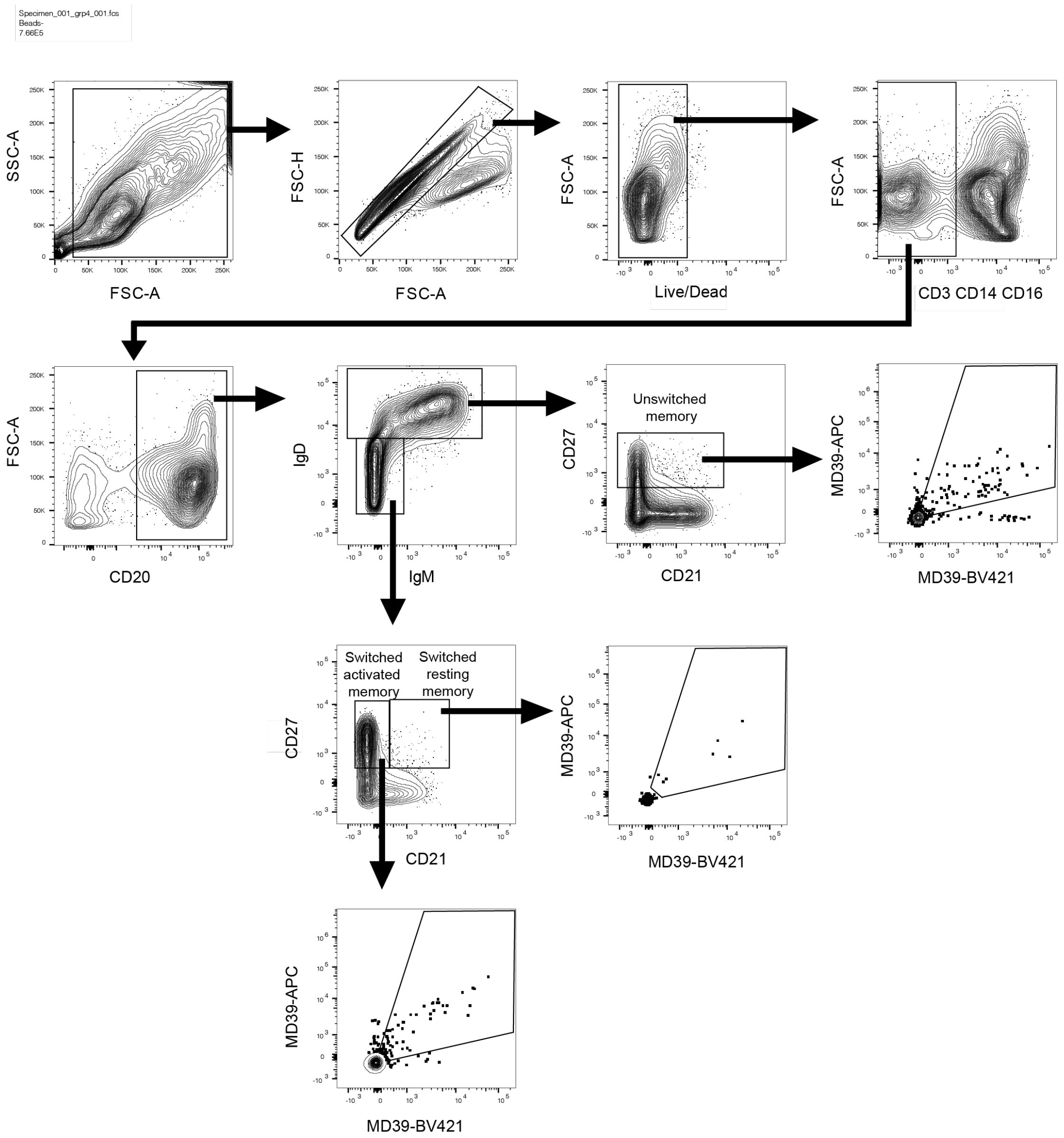

Figure S4: Flow cytometry gating strategy for analysis of antigen-specific memory B cells. PBMCs were collected at week 34 and immunolabeled for the markers indicated. Live cells (negative for Zombie Aqua), pre-gated on FSC-A/FSC-H to identify singlets, were negatively gated on CD3, CD14, and CD16 markers. Subsequent gating was applied to delineate unswitched, activated switched, and resting switched memory B cells, using markers CD20, IgM, IgD, CD21, and CD27. Lastly, memory B cells were selectively gated twice using MD39-tetramers to determine their antigen specificity.

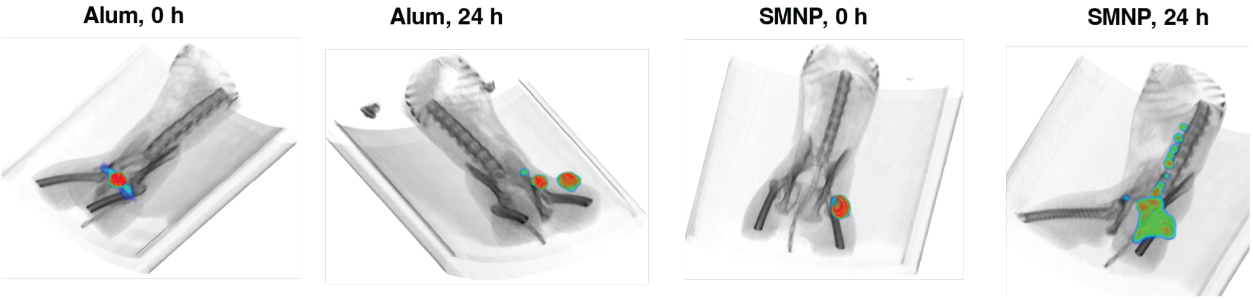

Figure S5: Trafficking of SMNP- and alum-adjuvanted MD39 immunogen to draining lymph nodes of macaques. Shown are representative 3D-rendered PET/CT images of the alum-adjuvanted immunization at 0 and 24 h post injection.

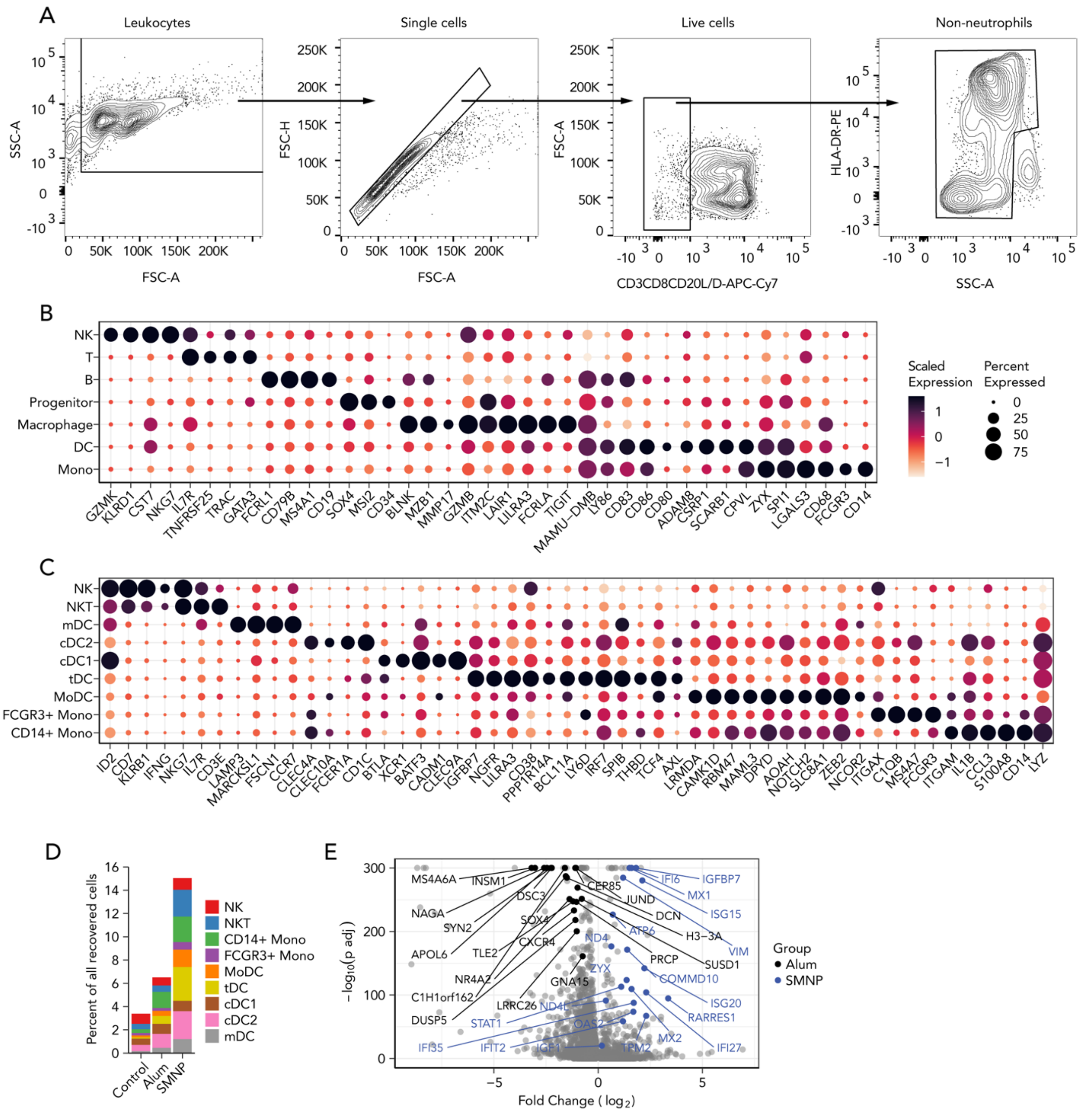

Figure S6: **SMNP creates an inflammatory environment in the LN**. (A) Flow cytometry gating strategy used for sorting out CD3^{low}, CD8^{low}, and CD20^{low} cells for scRNAseq. (B) Dot plot of gene signatures for each cell lineage. (C) Dot plot of gene signatures for the phenotypes of NK cells, DCs, and monocytes. For (B-C), the color of the dots indicates scaled expression levels, and the size of the dots represents the fraction of cells in the cluster that expresses the gene. (D) The percent of recovered cell phenotypes of NK cells, DCs, and monocytes from each group. (E) Volcano plot of differentially expressed genes between SMNP (blue) and alum (black) macrophages.

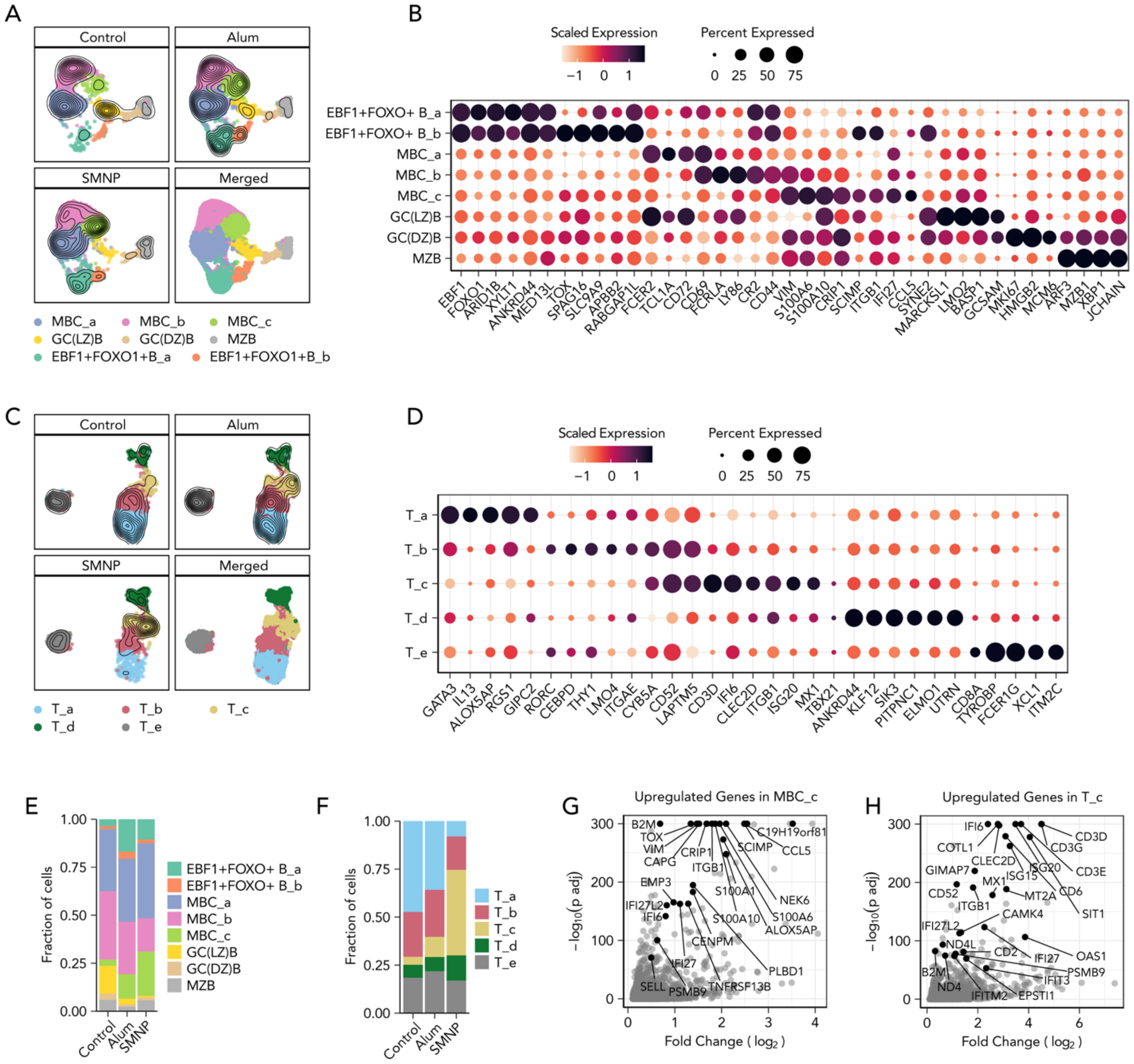

Figure S7. Transcriptional changes in T and B cell populations in response to alum vs. SMNP immunization. (A) UMAP of identified B cell phenotypic clusters. (B) Dot plot of gene signatures for each B cell phenotype. (C) UMAP of identified T cell phenotypic clusters. (D) Dot plot of gene signatures for each T cell phenotype. For (A & C), the contour lines represent the density of overlapping cells. For (B & D), the color of the dots indicates scaled expression levels, and the size of the dots represents the fraction of cells in the cluster that expresses the gene. (E) Distribution of identified B cell phenotypic clusters per group. (F) Distribution of identified T cell phenotypic clusters per group. (G) Volcano plot of significantly upregulated genes in MBC_c cluster compared to the rest of B cells. (H) Volcano plot of significantly upregulated genes in T_c cluster compared to the rest of T cells.

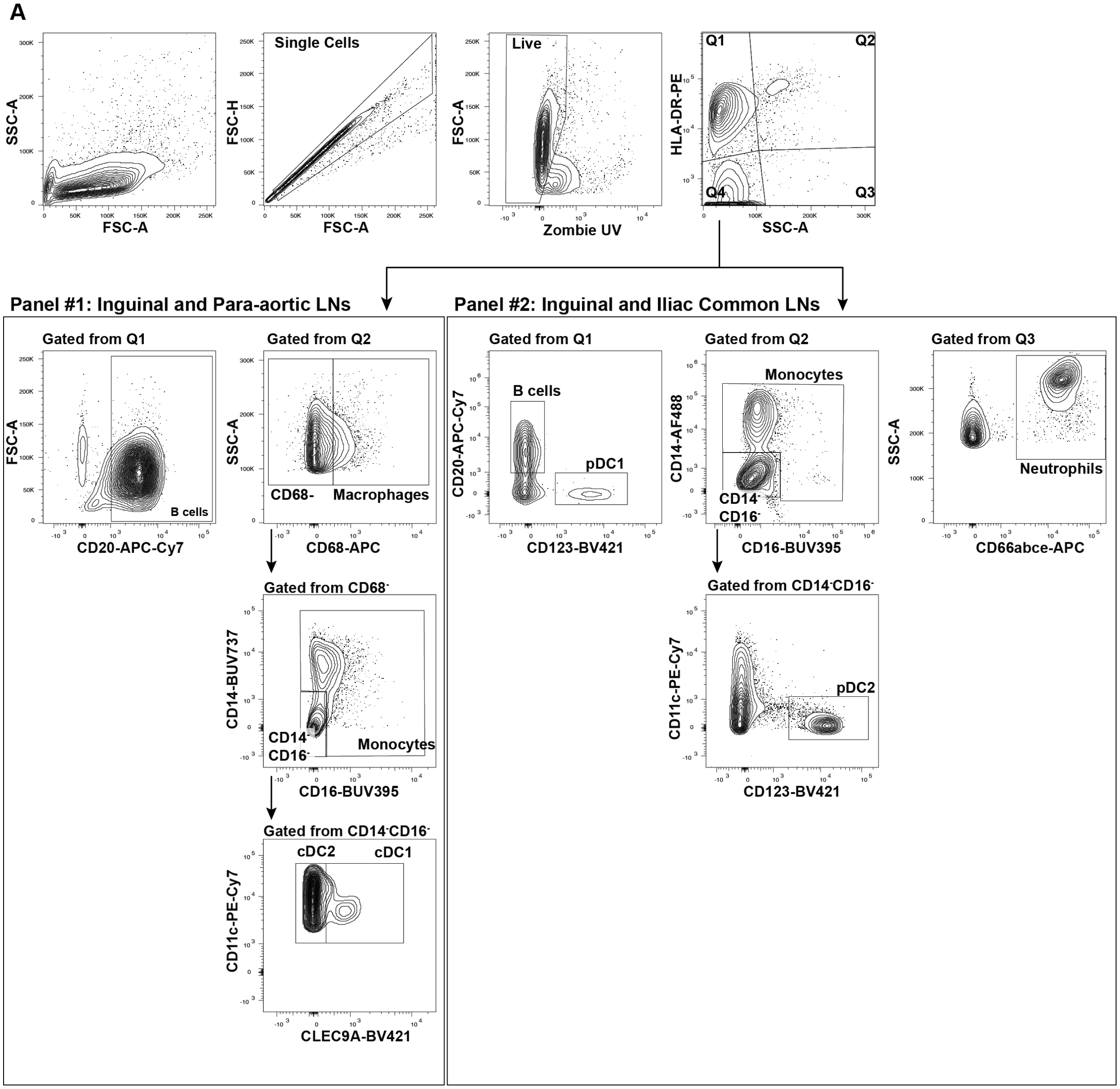

Figure S8: Flow cytometry gating strategy for analysis of myeloid cells and B cells in lymph nodes, Live cells (negative for Zombie Aqua), pre-gated on FSC-A/FSC-H to identify singlets, were gated on HLA-DR and SSC-A, and then on CD14, CD16, CD20, CD66, CD11c, CD68, and CLEC9A markers to delineate B cells, pDC1 and pDC2 cells, monocytes, neutrophils, macrophages, and cDC1 and cDC2 cells.

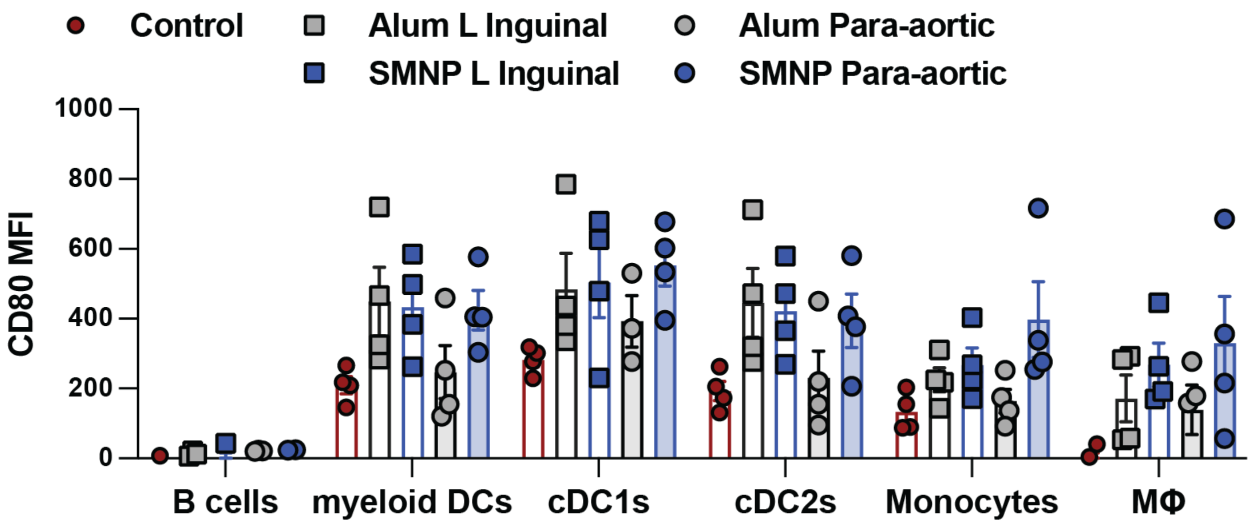

Figure S9. Innate immune responses to alum vs. SMNP immunization. CD80 expression on antigen presenting cells in proximal inguinal and distal para-aortic lymph nodes. Statistical analysis was done by Student's *t* test.
